# A non-natural nucleotide uses a specific pocket to selectively inhibit telomerase activity

**DOI:** 10.1371/journal.pbio.3000204

**Published:** 2019-04-05

**Authors:** Wilnelly Hernandez-Sanchez, Wei Huang, Brian Plucinsky, Nelson Garcia-Vazquez, Nathaniel J. Robinson, William P. Schiemann, Anthony J. Berdis, Emmanuel Skordalakes, Derek J. Taylor

**Affiliations:** 1 Department of Pharmacology, Case Western Reserve University, Cleveland, Ohio, United States of America; 2 The Wistar Institute Gene Expression and Regulation Program, Philadelphia, Pennsylvania, United States of America; 3 Department of Pathology, Case Western Reserve University, Cleveland, Ohio, United States of America; 4 Case Comprehensive Cancer Center, Case Western Reserve University, Cleveland, Ohio, United States of America; 5 Department of Chemistry, Cleveland State University, Cleveland, Ohio, United States of America; 6 Department of Biochemistry, Case Western Reserve University, Cleveland, Ohio, United States of America; The Rockefeller University, UNITED STATES

## Abstract

Telomerase, a unique reverse transcriptase that specifically extends the ends of linear chromosomes, is up-regulated in the vast majority of cancer cells. Here, we show that an indole nucleotide analog, 5-methylcarboxyl-indolyl-2′-deoxyriboside 5′-triphosphate (5-MeCITP), functions as an inhibitor of telomerase activity. The crystal structure of 5-MeCITP bound to the *Tribolium castaneum* telomerase reverse transcriptase reveals an atypical interaction, in which the nucleobase is flipped in the active site. In this orientation, the methoxy group of 5-MeCITP extends out of the canonical active site to interact with a telomerase-specific hydrophobic pocket formed by motifs 1 and 2 in the fingers domain and T-motif in the RNA-binding domain of the telomerase reverse transcriptase. In vitro data show that 5-MeCITP inhibits telomerase with a similar potency as the clinically administered nucleoside analog reverse transcriptase inhibitor azidothymidine (AZT). In addition, cell-based studies show that treatment with the cell-permeable nucleoside counterpart of 5-MeCITP leads to telomere shortening in telomerase-positive cancer cells, while resulting in significantly lower cytotoxic effects in telomerase-negative cell lines when compared with AZT treatment.

## Abstract 2

Telomerase is upregulated in the majority of all cancer cells, helping to drive tumorigenesis. This study describes the characterization of an artificial nucleoside analogue (5-MeCITP) that potently and selectively inhibits telomerase activity by binding to a unique pocket on the enzyme.

## Introduction

Linear chromosomes end in telomeres, which are repetitive G-rich sequences (TTAGGG in mammals) that guard against illicit DNA repair and end-to-end fusions [1,2]. Because replicative DNA polymerases operate directionally, they are unable to fully synthesize the end of the lagging strand of DNA [3]. Telomeres function to absorb the shortening that occurs at each cell division and, thus, protect against the loss of genetic information. The progressive shortening of telomeres restricts the number of times a cell will divide until a state of replicative senescence is induced, known as the Hayflick limit [4]. In highly proliferative tissues (e.g., during embryogenesis, germline and stem cells), telomerase, the enzyme that synthesizes telomere ends, is reactivated to extend telomere DNA to counterbalance telomere attrition [5]. Telomerase activity is undetectable in healthy somatic cells but is reactivated in 90% of all cancer cells, thereby driving tumorigenesis [6]. Because of this distinction, the inhibition of telomerase activity is a promising therapeutic strategy for many cancers.

Telomerase is a multicomponent, ribonucleoprotein enzyme that includes a catalytic telomerase reverse transcriptase (TERT) protein and a noncoding RNA molecule that serves as the template for synthesis of telomere DNA [7–9]. The genetic removal or pharmacological inhibition of telomerase results in sequential telomere shortening that eventually triggers senescence or apoptosis in cancer cells [10]. Among telomerase antagonists that have been developed, Imetelstat (GRN163L) is one of the most studied for its clinical application. Imetelstat is a lipid conjugated–modified oligonucleotide that is designed to impair telomerase activity by hybridizing with its template RNA [11]. A primary limitation of Imetelstat administration has been attributed to nonspecific interactions that are coordinated through the lipid moiety, which increases off-target effects [12]. Thus, the development of alternative small-molecule compounds that do not involve lipid conjugation will provide benefits for the administration of telomerase-dependent therapeutic approaches.

The use of soluble nucleoside analogs has provided a promising strategy for targeting reverse transcriptase (RT) enzymes that include telomerase. Nucleoside analogs represent a large class of compounds that have a successful history as antivirals and in cancer therapy [13,14]. Administered analogs enter cancer cells by nucleoside transporters, where they are phosphorylated by cellular nucleoside kinases to interfere with RNA and DNA synthesis or with nucleotide biogenesis [14,15]. As a primary example, azidothymidine (AZT) is a thymidine analog with clinical success as an RT inhibitor, specifically in the treatment of human immunodeficiency virus (HIV-1) infections [16,17]. Although TERT contains additional domains, its catalytic core is structurally homologous to the RT domain of HIV-1 [18]. Therefore, it is not surprising that nucleoside reverse transcriptase inhibitors (NRTIs) exhibit inhibitory properties against telomerase as well [19–21]. Indeed, AZT treatment has reached Phase II clinical trials as a telomerase inhibitor for the treatment of colorectal cancer [22], pancreatic adenocarcinoma [23], and other malignant tumors [24,25]. Although promising, AZT treatment exhibits significant cytotoxic effects, as it indiscriminately inhibits other DNA polymerases as well [26,27].

Here, we have investigated a set of chemically diverse nucleotide analogs as potential inhibitors of telomerase activity. In doing so, we have identified 5-methylcarboxyl-indolyl-2′-deoxyriboside 5′-triphosphate (5-MeCITP) as an inhibitor of telomerase activity. Structural studies reveal that the analog binds to the TERT active site with the nucleobase inverted so that the methylcarboxyl modification interacts with a telomerase-specific, hydrophobic pocket adjacent to the TERT active site that is formed by motifs 1 and 2 within the fingers domain and the telomerase-specific motif (T-motif) in the telomerase RNA-binding domain (TRBD). The unconventional positioning of 5-MeCITP, along with an inability to form proper Watson-Crick base pairings, results in the displacement of the complementary bases of the RNA template away from the TERT active site. Administration of the cell-permeable form of 5-MeCITP (5-MeCIdR) results in telomerase-dependent telomere shortening in cancer cells. Cumulatively, we identify a nucleotide analog that implements a unique mechanism to selectively inhibit telomerase-mediated extension of telomere DNA.

## Results

### Screening of indolyl-2′-deoxynucleotide analogs for telomeric incorporation

**Fig 1:**
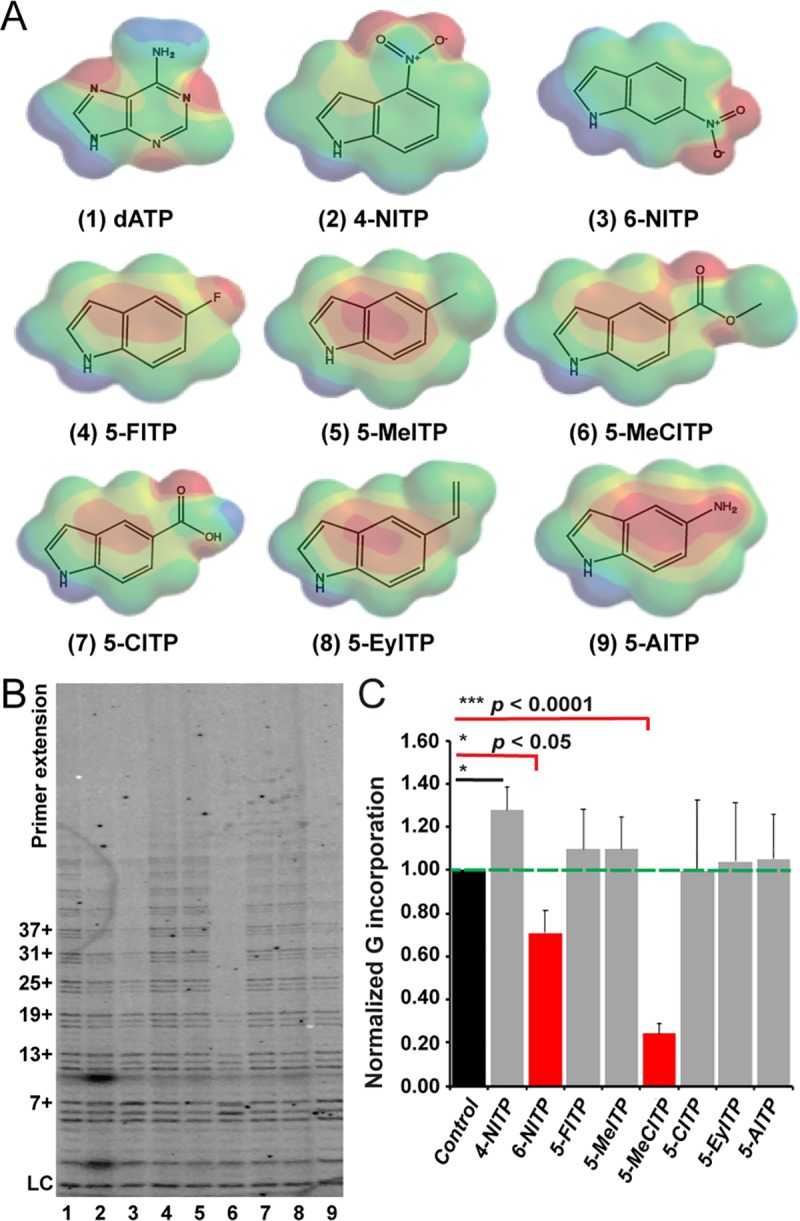
Nucleotide 5-MeCITP is an inhibitor of telomerase activity. (**A**) Chemical structures and electron density surface potentials of indolyl nucleotide analogs used in this study. For clarity, only nucleobase structures are shown. Abbreviations at the bottom of each nucleobase correspond to the full name of the nucleotide as described in S1 Table. (**B**) Screening of nucleotide analogs via the direct telomerase activity assay reveals that 5-MeCITP reduces telomerase activity by 80% compared with untreated control. Numbers at the bottom of the gel correspond to the analog number indicated in panel A. Control refers to untreated reaction. Values to the left of the gel depict the number of deoxynucleotides added by telomerase to the primer d(GGGTTA)_3_. (**C**) Quantitation of G (dGTP) incorporation relative to control for samples analyzed in panel B. Error bars indicate the standard deviation calculated from three replicates. A two-tailed Student *t* test was used to determine *p*-values. *p < 0.05, ***p < 0.0001. Data associated with this figure can be found in the supplemental data file (S1 Data). dATP, deoxyadenosine triphosphate; dGTP, deoxyguanosine triphosphate; LC, loading control; 5-MeITP, 5-methylindolyl-2′-deoxyriboside 5′-triphosphate; 4-NITP, 4-nitroindolyl-2′-deoxynucleoside 5′-triphosphate; 6-NITP, 6-nitroindolyl-2′-deoxynucleoside 5′-triphosphate; 5-AITP, 5-aminoindolyl-2′-deoxyriboside 5′-triphosphate; 5-CITP, 5-carboxylindolyl-2′-deoxyriboside 5′-triphosphate; 5-EyITP, 5-ethyleneindolyl-2′-deoxyriboside 5′-triphosphate; 5-MeCITP, 5-methylcarboxyl-indolyl-2′-deoxyriboside 5′-triphosphate.

Telomerase functions as a unique RT that uses a specialized, noncoding RNA as a template for telomere DNA synthesis [9,18,28,29]. This distinctive trait, along with additional differences in sequence, structure, and overall function compared with conventional replicative and repair polymerases [30,31], could provide key features of telomerase that can be exploited therapeutically. Pursuing this potential, we selected a set of indolyl-2′-deoxynucleotide analogs that exhibit different chemical properties and screened them for their ability to inhibit telomerase activity (**Fig 1A** and **S1 Table**). These analogs were designed to mimic the core structure of deoxyadenosine triphosphate (dATP), but with the introduction of various functional groups at the 5- or 6-position of the indole ring to systematically modulate biophysical features such as hydrophobicity, size variation, shape, and π-electron density that might contribute to selective interactions with telomerase. Modification of the nucleobase at these positions will additionally disturb proper Watson-Crick base pairing with the RNA template. Finally, the individual analogs investigated are already known to exhibit differential effects on diverse DNA polymerases that include HIV-1 RT, bacteriophage T4 DNA polymerase, and *Escherichia coli* Klenow fragment [32–36].

Alterations that these artificial nucleotide analogs had on telomerase activity were characterized using a modified direct in vitro telomerase activity assay [37]. Our first set of experiments were designed to determine whether any of the analogs serve as effective substrates for telomerase-mediated extension. As such, the indolyl analogs were used in place of the structurally similar native dATP nucleotide, along with deoxythymidine triphosphate (dTTP) and deoxyguanosine triphosphate (dGTP). After a 30-minute incubation period, the ability of telomerase to incorporate the nucleotide analogs into a telomere DNA product was evaluated by analyzing the extended products and comparing with those generated with dATP as the nucleotide substrate (**S1 Fig**). Extension of DNA products was negligible under these conditions, as product lengths were comparable to those measured in the complete absence of dATP and nucleotide analog (**S1 Fig**). These data suggest that telomerase cannot incorporate any of the analogs into telomere DNA products at the concentration tested or that selective incorporation of the indolyl analogs by telomerase causes chain termination to produce very short products.

### Nucleotide 5-MeCITP inhibits telomerase activity in vitro

We next performed the direct in vitro telomerase activity assay in the presence of native deoxynucleoside triphosphates (dNTPs) along with each indolyl-2′-deoxynucleoside triphosphate analog. For initial experiments, native dATP concentrations were maintained near the predetermined relative rate constant (*K*_1/2_) value of approximately 1 μM for the assay (**S2 Fig**) and in the presence of 200 μM nucleotide analog for a single reaction conducted at the pre-steady-state condition of 30 minutes (**S2 Fig and Fig 1B**). Extended products were analyzed to conclude that the addition of 6-nitroindolyl-2′-deoxyriboside 5′-triphosphate (6-NITP) resulted in a slight but significant (*p* < 0.05) decrease in telomerase activity (**Fig 1C**). Strikingly, 5-MeCITP produced a greater than 80% reduction in telomerase activity under identical conditions (*p* < 0.0001) (**Fig 1C**). None of the other analogs investigated behaved as inhibitors against telomerase activity under these experimental conditions, including 5-methylindolyl-2′-deoxyriboside 5′-triphosphate (5-MeITP) and 5-carboxylindolyl-2′-deoxyriboside 5′-triphosphate (5-CITP), which are structurally similar to 5-MeCITP (**Fig 1A and S1 Table**). These findings indicate that the combination of methyl and carboxyl moieties is required for effective inhibition of telomerase by 5-MeCITP.

**Fig 2:**
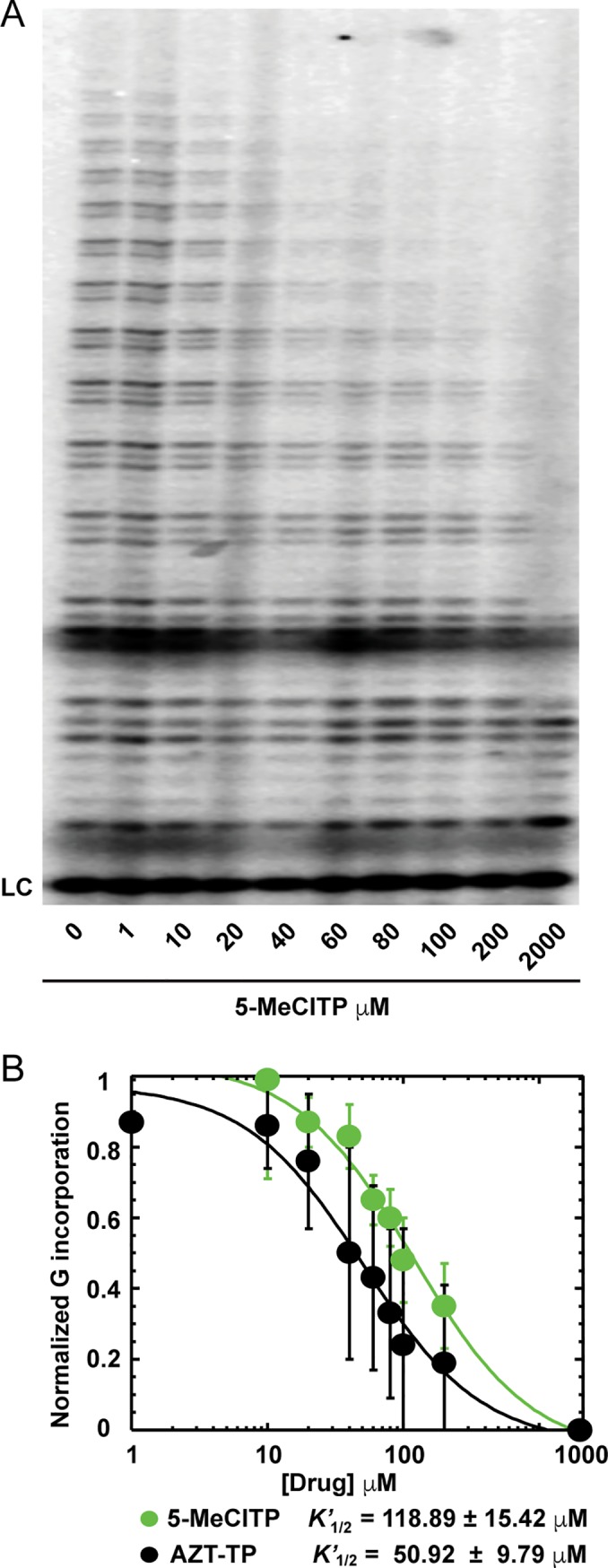
Nucleotides 5-MeCITP and AZT-TP inhibit telomerase in a dose-dependent manner. Direct telomerase extension assay with increasing concentrations of (**A**) 5-MeCITP (0–2 mM) and (**B**) AZT-TP (0–2 mM) reveal inhibition of telomerase activity in a dose-dependent manner. Activity (normalized G incorporation) was quantified and plotted against inhibitor concentration to determine relative *K’*_1/2_ values for each compound (*n* = 4). Data associated with this figure can be found in the supplemental data file (S1 Data). AZT-TP, azidothymidine triphosphate; G, deoxyguanosine triphosphate; LC, loading control; 5-MeCITP, 5-methylcarboxyl-indolyl-2′-deoxyriboside 5′-triphosphate.

To better define the impact of 5-MeCITP on telomerase activity, we compared its inhibitory effects with that of AZT triphosphate (AZT-TP). For these experiments, telomerase activity was measured in the presence of increasing inhibitor concentrations in a dose-dependent manner (**Fig 2**). The data were fit to calculate apparent rate constants (*K’*_1/2_) for each compound that describes the concentration of inhibitor in which telomerase activity is 50% as compared with untreated controls. These constants were determined to be 118.89 ± 12.42 and 50.92 ± 9.79 μM for 5-MeCITP and AZT-TP, respectively, under these experimental conditions (**Fig 2** and **S3 Fig**).

To probe the mode of binding of 5-MeCITP to telomerase, another experiment was performed using the direct telomerase assay in the presence of 400 μM 5-MeCITP and with increasing concentrations of dATP. Fitting of the data revealed a nearly complete recovery of telomerase activity at elevated concentrations of dATP (**S4 Fig**). The *K*_1/2_ for dATP increased by approximately four times when the experiments were performed in the presence of 5-MeCITP (**S4 Fig**). Cumulatively, these results suggest that 5-MeCITP inhibits telomerase activity by competing with dATP binding.

### The noncanonical binding of 5-MeCITP disturbs telomerase RNA template base positioning

**Fig 3:**
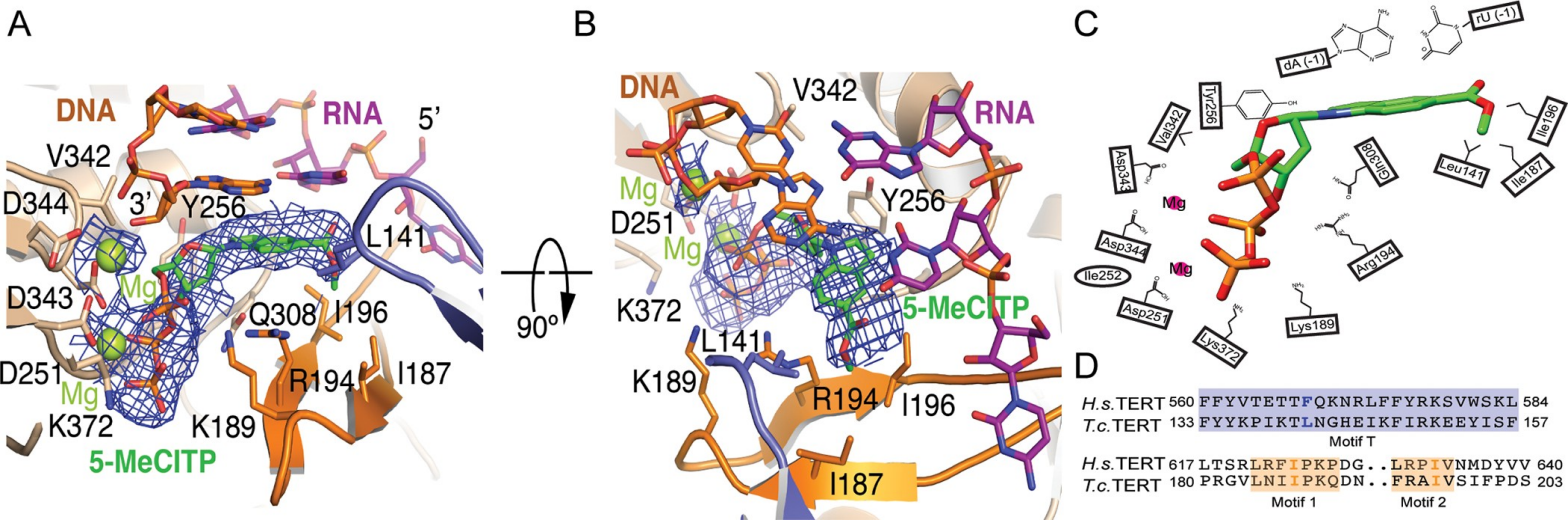
Nucleotide 5-MeCITP occupies the telomerase deoxynucleotide binding site and displaces the RNA template base. (**A**) Structure of *T*. *castaneum* TERT in complex with a hybrid RNA-DNA and 5-MeCITP. The RNA-binding domain is shown in blue, the finger domain in orange, and the palm domain in yellow. The RNA template strand is shown in purple and the DNA strand in orange. (**B**) Panel (A) rotated 90°. (**C**) Two-dimensional diagram of the molecular interactions of 5-MeCITP with *T*. *castaneum* TERT. (**D**) Sequence alignment reveals sequence comparison of residues in the T-motif, and motifs 1 and 2 of *Homo sapiens* and *Tribolium castaneum* TERT. TERT, telomerase reverse transcriptase; 5-MeCITP, 5-methylcarboxyl-indolyl-2′-deoxyriboside 5′-triphosphate.

To identify the precise interactions that govern 5-MeCITP binding to telomerase, we crystalized the *T*. *castaneum* TERT-MeCITP complex along with a nucleotide hairpin that functionally mimics the telomerase RNA template-telomere DNA substrate [18]. The structure of the complex refined to 2.8 Å identifies density corresponding to an unpolymerized 5-MeCITP residing in the active site of the enzyme (**Fig 3** and **S2 Table**). The triphosphate group of 5-MeCITP is coordinated by two metal ions in a manner that is structurally similar to that of a nonhydrolyzable nucleotide analog bound to the active site of other polymerases [38–40]. The modified nucleobase of 5-MeCITP adopts a noncanonical configuration in which it is rotated 180° as compared with the expected orientation of a native dNTP forming Watson-Crick base pairs with the RNA template (**S5 Fig**). This configuration is very likely governed by an inability to properly base pair with RNA combined with selective interactions formed between the methylcarboxyl moiety of 5-MeCITP and TERT. Specifically, the methylcarboxyl moiety occupies a hydrophobic patch formed between motifs 1, 2 of the fingers domain and T-motif in the TRBD. The positioning of the methylcarboxyl group of 5-MeCITP is coordinated by interactions with Ile187 and Ile196 of motifs 1 and 2 and with Leu141 of the T-motif of *T*. *castaneum* TERT (**Fig 3A–3C**). The unique positioning of the modified nucleotide coincides with a displacement of the nucleobase of the template away from its canonical position (**S5 Fig**). It is worth noting that the RNA base displacement is likely to be subtle in the context of the full-length RNA, where additional protein-RNA contacts within telomerase stabilize this interaction [41]. Nonetheless, an overlay of the available TERT structures of *T*. *castaneum* [18], *Homo sapiens* [42], and *Tetrahymena thermophila* [43] highlights a structural arrangement that is relatively conserved among species (**S6 Fig)**. Similarly, the interaction with the methylcarboxyl moiety of the artificial nucleotide in *T*. *castaneum* TERT is coordinated by two isoleucine residues located in motifs 1 and 2 that are conserved in the human enzyme (**Fig 3D**). The methylcarboxyl group of 5-MeCITP is coordinated by a leucine in *T*. *castaneum* and a phenylalanine in *H*. *sapiens*, residing in T-motif of TERT. While either amino acid would be expected to make similar hydrophobic contributions to the pocket that is formed, the presence of a bulkier phenylalanine in human TERT might provide a tighter pocket for 5-MeCITP to bind to or require a structural rearrangement to accommodate the methylcarboxyl moiety. In any event, an analogous hydrophobic pocket is very likely formed in human telomerase to trap 5-MeCITP in a similarly inactive state.

The co-crystal structure shows that 5-MeCITP does not form direct interactions with the RNA template, suggesting that inhibition is not specific to incorporation of a specific dNTP. Rather, 5-MeCITP likely prevents access of all dNTPs to the active site of telomerase. To test this hypothesis, we performed the direct telomerase incorporation assay with 5-MeCITP and human telomerase along with dATP, dTTP, or dGTP at limiting concentrations in the reaction. As anticipated, 5-MeCITP displayed similar inhibitory properties under all three conditions (**S7 Fig**). These results further suggest that the inhibition of telomerase by 5-MeCITP is not specific against a particular dNTP substrate, but that it competes with all dNTPs for access to the telomerase active site.

### Nucleoside 5-MeCIdR leads to telomere shortening in a telomerase-dependent manner and is less toxic than AZT

**Fig 4:**
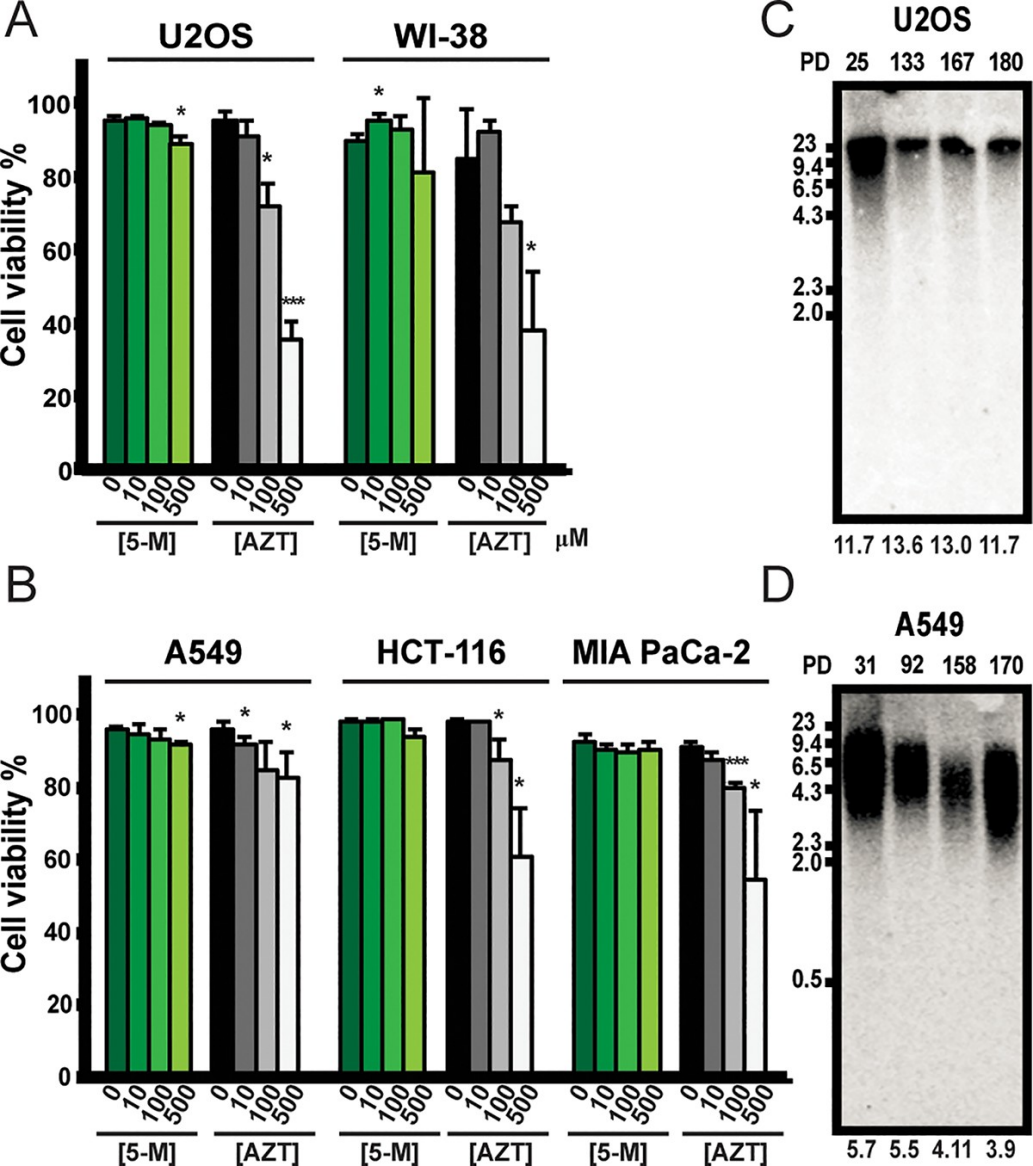
Nucleoside 5-MeCIdR leads to telomere shortening in telomerase-positive cells selectively. Trypan exclusion assay for telomerase-negative (**A**) and telomerase-positive cells (**B**) shows that 5-MeCIdR (abbreviated as 5-M, green scale bars) treatment is less toxic than AZT (gray scale bars) to all treated cells. *p < 0.05, ***p < 0.001. Error bars indicate the standard deviation values from three replicate experiments. (**C**) Treatment of telomerase-negative U2OS cells with 100 μM 5-MeCIdR does not alter telomere length, while telomeres in telomerase-positive A549 cells (**D**) get shorter with increasing population doublings (PDs). Numbers at the bottom of the Southern blots indicate the average telomere lengths. Data associated with this figure can be found in the supplemental data file (S1 Data). AZT, azidothymidine; PD, population doubling; 5-M/5-MeCIdR, cell-permeable nucleoside form of 5-MeCITP.

We next characterized the growth properties of different cell lines that were treated with 5-MeCIdR, the cell-permeable nucleoside counterpart of 5-MeCITP. These studies used a panel of genetically distinct cell types that included A549 (lung cancer), HCT-116 (colon cancer), and MIA PaCa-2 (pancreatic cancer) cell lines that are telomerase positive, and U2OS (osteosarcoma) cells, which implement alternative lengthening of telomeres (ALT) instead of telomerase for maintaining telomere length. Finally, WI-38 (WI-38 VA13 subline) cells were used as human lung fibroblast cells that do not exhibit ALT or telomerase activity. In general, treatment with 5-MeCIdR (10–500 μM) for up to 3 days did not produce any adverse effects on cell viability for any of the five cell lines investigated (**Fig 4A** and **4B**; **S8 Fig**). In contrast, AZT-treated cells exhibited lower cell survival in both telomerase-positive and telomerase-negative cell lines, especially at the elevated doses (>100 μM) (**Fig 4A** and **4B**; **S8 Fig**).

We next monitored the effects of long-term exposure of cells to 5-MeCIdR (100 μM) on telomere length maintenance. Telomerase-negative U2OS cells treated with 5-MeCIdR maintained a constant telomere length distribution even after 180 population doublings (**Fig 4C**). In contrast, telomere length became progressively shorter in telomerase-positive A549, HeLa, and HCT116 cells treated with 5-MeCIdR and compared with DMSO control (**Fig 4D** and **S9 Fig**). Consistent with these data, long-term administration of 5-MeCIdR resulted in a significant increase in senescence-associated β-galactosidase activity in telomerase-positive cancer cells as compared with DMSO-treated control cells (**S10 Fig**). In summary, these data indicate that 5-MeCIdR treatment selectively leads to telomere shortening and initiates senescence in telomerase-positive cancer cells.

## Discussion

As most tumors rely on telomerase to drive cellular immortality, the development of compounds designed to selectively impede telomerase activity remains a promising therapeutic strategy. In this study, we tested the ability of a set of indolyl nucleotide analogs that display diverse physicochemical properties to impede telomerase function. These results, and especially the identification of 5-MeCITP as an inhibitor of telomerase, provide exciting potential for further drug development.

While used with other antiviral agents in the treatment of HIV-1, the administration of AZT is associated with enhanced cytotoxicity in healthy cells, which has limited its clinical potential [44–48]. Here, we report that 5-MeCITP inhibits telomerase activity with a potency similar to that of AZT-TP. Compared with AZT, however, 5-MeCIdR administration was more tolerated by cells and produced minimal changes in cell viability. Together, these data indicate that 5-MeCIdR treatment is generally more tolerated by cells than AZT, potentially due to higher selectivity that limits off-target effects on metabolic pathways and non-telomerase polymerases.

Our results also provide essential and fundamental knowledge regarding the selectivity of small-molecule interactions with the telomerase catalytic site. For example, the structure of 5-MeCITP bound to *T*. *castaneum* TERT reveals that the carboxyl group helps to position the methyl moiety at a distance away from the ribose ring so that the methyl can fit into a specific hydrophobic pocket of TERT, while the phosphate groups and ribose ring occupy a native-like position in the active site of the enzyme. This finding could explain why the active site of telomerase is more selective for 5-MeCITP over the other nucleotide analogs we investigated, a notion that is supported by docking experiments. In this set of experiments, none of the other analogs were predicted to bind in a manner such that the modifications within the nucleobase could access the hydrophobic pocket formed by the T-motif and motifs 1 and 2 while concomitantly occupying the nucleotide binding pocket of TERT (**S11 Fig**). As such, it is very likely that the carboxyl group of 5-MeCITP merely serves as an extension to place the aliphatic methyl moiety in the vicinity of the hydrophobic pocket of TERT so that it may form favorable interactions there.

As the specific, physicochemical properties of 5-MeCITP facilitate its interactions with telomerase, the elements that form the unique binding pocket of telomerase might similarly explain the selective nature of 5-MeCITP binding to telomerase over other polymerases. Interestingly, the hydrophobic binding pocket of telomerase is formed by regions that are specific to telomerase (**S12 Fig**). The T-motif is a conserved structure that is unique to TERT proteins. It is postulated to interact with the template boundary element (TBE) [41] located just upstream of the RNA template to help mediate positioning of the RNA template to guide nucleotide selection and facilitate telomerase processivity [49–51]. Motifs 1 and 2 form an insertion within the fingers subdomain and are conserved among the RT family, including TERTs [7,29]. Interestingly, 5-MeCITP also inhibits HIV-1 RT activity [32]. Therefore, it is predicted that motifs 1 and 2 provide the more favorable interactions to promote 5-MeCITP binding to HIV-1 RT or TERT active sites and that contributions from the TERT-specific T-motif play a less prominent role in the selective binding of this analog. In contrast to HIV-1 RT and TERT enzymes, other DNA polymerases lack the analogous environment formed by motifs 1 and 2 in the active site and coincidentally lack the putative hydrophobic pocket that facilitates interactions of 5-MeCITP with telomerase (**S12 Fig**). As a result, 5-MeCITP does not inhibit DNA polymerases such as T4 DNA polymerase and *E*. *coli* Klenow fragment. Instead, these polymerases can effectively incorporate 5-MeCITP, at least at positions that are opposite abasic sites in the template DNA [32,36].

**Fig 5:**
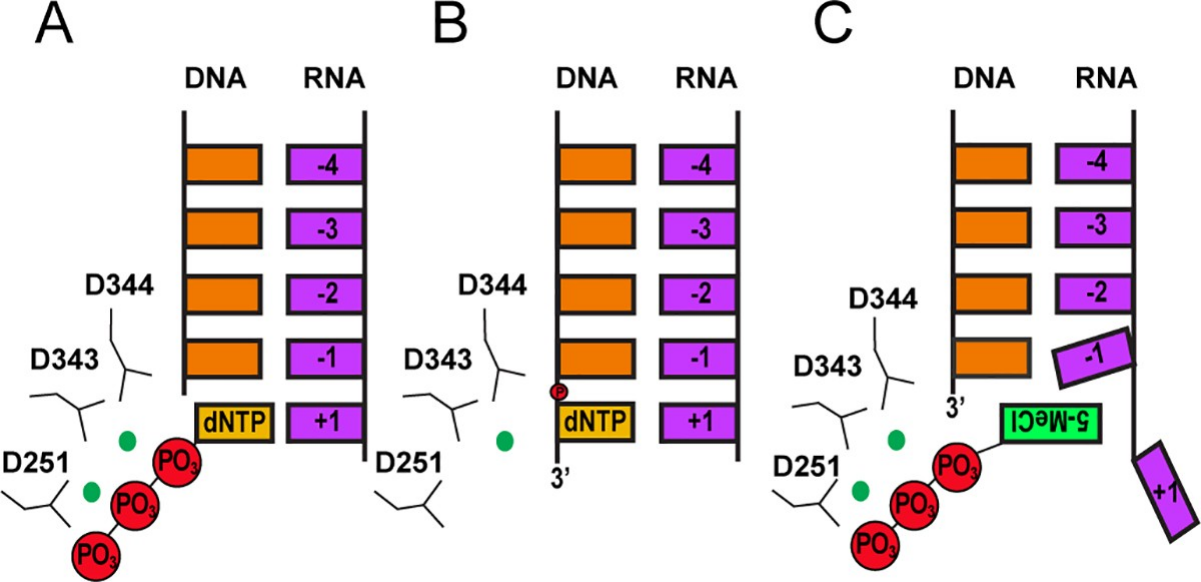
Proposed mechanism of action of 5-MeCITP for inhibiting telomerase activity. (**A**) Pre-polymerization state of a natural telomerase substrate (dNTP). The two magnesium ions help to coordinate the nucleophilic attack of the 3′-OH of the terminal dNTP of the telomeric DNA strand on the α-phosphate group of the dNTP in the TERT active site. (**B**) Post-polymerization state of a natural telomerase dNTP substrate. The dNTP forms a Watson-Crick base pair with the RNA template to aid in nucleophilic attack of the phosphate backbone for incorporation into the synthesized DNA strand. (**C**) 5-MeCITP does not base pair appropriately with the RNA template, thereby occluding the RNA from the active site. As a consequence, 5-MeCITP is not incorporated into the DNA chain. dNTP, deoxynucleotide triphosphate; TERT, telomerase reverse transcriptase; 5-MeCITP, 5-methylcarboxyl-indolyl-2′-deoxyriboside 5′-triphosphate.

The X-ray crystal structure of *T*. *castaneum* TERT bound with an RNA template mimic and a complementary telomeric single stranded DNA (ssDNA) has a 3′ deoxyguanosine (dG) nucleotide occupying the catalytic site [18]. That particular structure represents a post-polymerization state in which the terminal dG is covalently bound to the synthesized ssDNA and its lone backbone phosphate group is coordinated by a single Mg^2+^ ion. In contrast, the structure presented here reveals that 5-MeCITP is not incorporated into the DNA strand and instead binds to the *T*. *castaneum* TERT active site with the triphosphate coordinated by a pair of Mg^2+^ ions (**Fig 3**). The α-phosphate of 5-MeCITP is situated at a distance that is approximately 5 Å from the nucleophilic 3′-hydroxyl group of the terminal dATP of the ssDNA that is being extended and is therefore too far for nucleophilic attack and incorporation into the 3′ end of the DNA. Additionally, the inability of 5-MeCITP to properly base pair with the RNA template most likely prevents the nucleotide analog from adopting a position that would facilitate polymerization into the ssDNA chain. Without base pairing, the nucleobase of 5-MeCITP forms alternative interactions in the TERT active site. Specifically, our data reveal that it flips so that the methylcarboxyl group can selectively form hydrophobic interactions with the unique pocket formed by the T-motif and motifs 1 and 2 of TERT. This unorthodox positioning in the TERT active site further perturbs the complementary RNA base away from the active site (**Fig 5**). In summary, the inability of the modified nucleotide to form proper Watson-Crick base pairs with RNA combined with the favorable and unique interactions formed with TERT are important contributors to 5-MeCITP’s unique mechanism of action. The structure-function studies reported here therefore provide an opportunity to develop additional analogs to exploit these properties and to develop more selective and potent telomerase inhibitors.

## Methods

### Nucleotide analogs

Nucleotide analogs 4-NITP [33], 6-NITP [33], 5-FITP [34], 5-MeITP [35], 5-MeCITP [36], 5-CITP [32], 5-EyITP [35], and 5-AITP [34] were characterized and synthesized as previously described. AZT-TP was purchased from Trilink Biotechnologies.

### Direct telomerase incorporation assay

Telomerase activity assays were performed as described previously [37]. Briefly, 2 μL of hTR and hTERT-transfected HEK 293T cell lysate (super telomerase cell extracts) were used in each 20-μL reaction. Unless otherwise noted, all assays were conducted with a mixture containing 35 mM Tris-HCl, pH 8.0, 0.7 mM MgCl_2_, 1.8 mM β-mercaptoethanol, 0.7 mM spermidine, 35 mM NaCl, 100 μM dTTP, 100 μM dATP, 2.9 μM dGTP, 0.33 μM [α-^32^P]-dGTP (10 μCi/μL, 3000 Ci/mmol, Perkin–Elmer), and 1 μM 18-nt substrate d(GGGTTA)_3_ primer. Each reaction was carried out for 30 minutes at 30°C before quenching with 100 μL of a quench buffer containing 3.6 M NH_4_OAc, 300 μM glycogen, and 400 μM EDTA. In each reaction, a 5′-^32^P–labeled hT18 primer (GGGTTA)_3_ was used as a loading control. All ssDNA products were ethanol precipitated and analyzed on a 12% polyacrylamide/7 M urea/1X Tris-Borate-EDTA denaturing gel. The gels were dried and exposed overnight to phosphorimager plates, which were imaged on a Typhoon FLA 9500 bimolecular imager (GE Healthcare), and densitometry was performed by SAFA footprinting software [52]. Quantification of telomerase assay products (normalized G incorporation) were used to determine total enzyme activity for each reaction by quantifying the signal of all dGTPs incorporated into the extended products, according to Eq 1.

$$G\;incorporation=\sum \frac{Norm.R_{f}*Reference\;LC}{LC\;of\;the\;lane}$$ (1)

where *Norm*. *R*_*f*_ refers to the normalization of the radioactivity factor and is obtained by dividing the raw intensity values by the number of Gs added to the extended primer. LC of the lane refers to loading control for each individual lane, while Reference LC refers to a common loading control used for normalization between lanes. Dose response curves were generated by plotting the G incorporation value against the concentration of the drug being added.

### Direct telomerase incorporation assay to determine *K*_1/2_

The *K*_1/2_ determination for each dNTP was conducted under the standard telomerase extension assay conditions described in the direct telomerase incorporation assay in the Methods section, but with increasing concentrations of that particular dNTP that ranged from 0–100 μM. Normalized G incorporation was plotted against that dNTP concentration. Normalized G incorporation was calculated by following Eq 1, and each reaction was then normalized as indicated in each figure. Normalized G incorporation was plotted against deoxynucleotide (dATP or dTTP) concentration to generate a dose-response curve that was fit by a hyperbolic equation using Kintek Explorer 7.6 software [53] and KaleidaGraph.version 3.5. The hyperbolic equation used to fit the data is described in Eq 2:

$$Normalized\;G\;incorporation=a*\frac{\left[dNTP\right]}{K_{1/2}+[dNTP]}+c$$ (2)

where *a* is the amplitude, *c* is the offset, and [*dNTP*] is the deoxynucleotide triphosphate concentration that is changing (dATP or dTTP).

For the experiments of substrate competition between dATP and 5-MeCITP, the telomerase assay was performed with 400 μM 5-MeCITP and increasing concentrations of dATP that ranged from 0 to 100 μM. The quantitation and fitting of this data was done as explained above using Eqs 1 and 2. To determine whether 5-MeCITP blocked incorporation of dTTP, dATP, or dGTP, the telomerase assay was performed under standard conditions with differing dNTP concentrations. To determine whether 5-MeCITP blocked incorporation of dTTP, the dNTP concentrations were 3 μM dGTP (including 10% [α-^32^P]-dGTP), 2 μM dTTP, 100 μM dATP, and 0 to 400 μM 5-MeCITP. To determine whether 5-MeCITP blocked incorporation of dGTP, the dNTP concentrations were 3 μM dGTP, 2 μM dTTP that included 2 μL [α-^32^P]-dTTP (10 μCi/μL, 3000 Ci/mmol, Perkin–Elmer), 100 μM dATP, and 0 to 400 μM 5-MeCITP.

### Direct telomerase incorporation assay to determine *K’*_*1/2*_ of AZT and 5-MeCITP

For assays involving 5-MeCITP, concentrations of dTTP were maintained at 100 μM and dATP at a concentration near its determined *K*_1/2_ value of 1 μM, along with increasing concentrations of 5-MeCITP that ranged from 0 to 2 mM. The observed *K’*_*1/2*_ of AZT-TP was determined in a similar manner but with dTTP used at a limiting concentration near its *K*_1/2_ value of 2 μM and dATP at 100 μM. For these reactions, increasing concentrations of AZT-TP (0–2 mM) were added to each reaction at the specified dose. Normalized G incorporation was calculated following Eq 1, and each reaction was then normalized to the reaction with no inhibitor or as indicated in each figure. Normalized G incorporation was plotted against inhibitor concentration to generate a dose-response curve that was fit by a hyperbolic equation using Kintek Explorer 7.6 software [53] and KaleidaGraph.version 3.5. The hyperbolic equation used to fit the data is described in Eq 3:

$$Normalized\;G\;incorporation=a*\frac{\left[i\right]}{K_{1/2}^{\prime }+[i]}+c$$ (3)

where *a* is the amplitude, *c* is the offset, and [*i*] is the inhibitor concentration. This fitting generated an observed affinity constant rate value (*K*’_1/2_) that describes the inhibitor concentration needed to observe half of the normalized G incorporation.

### Cell culture

Human HCT116, MIA PaCa-2, A549, HeLa, WI-38, and U2OS cell lines were obtained from ATCC. All cell lines were grown in Dulbecco’s Modified Eagle Medium (DMEM) supplemented with 10% fetal bovine serum (FBS) at 37°C with 5% CO_2_. For viability studies, cells were treated with a final concentration of 100 μM 5-MeCIdn in the presence of 0.0003% DMSO or with 0.0003% DMSO alone. Every 4 days cells were provided fresh media containing analog/DMSO or DMSO alone. Every 7 days, cells were counted, and 5.0 × 10^4^ cells were transferred into a new plate with fresh media containing nucleotide analog/DMSO or DMSO. For generation of growth curves, the cells were treated with the indicated drugs for 5 days and trypsinized. Viable cells were identified by trypan blue exclusion and counted on a hemocytometer.

### Telomere restriction fragment analysis

Telomere restriction fragment (TRF) analysis was performed using a commercial kit using standard protocols (TeloTAGGG Telomere Length Assay, Catalog no. 12209136001, Roche Diagnostics Corporation, Indianapolis, IN). DNA was extracted from A459, HCT116, MIA-Pa-Ca-2, Wi-38, and U2OS cells after 6 months’ treatment with 100 μM of 5-MeCIdR/DMSO or DMSO alone. A total of 2 μg DNA was digested overnight with Rsa I and Hinf I at 37°C and electrophoresed through 0.8% agarose gels in 1 × TAE at 50 V for 4 hours. Gels were denatured and neutralized prior to capillary transfer overnight. Telomeric DNA was transferred onto a Hybond-N^+^ membrane (GE Healthcare, Chicago, IL) using 20× SSC buffer. The transferred DNA was fixed by UV cross-linking. The cross-linked membrane was then hybridized with a ^32^P-labeled synthetic telomere probe with the sequence (GGGTTA)_4_ overnight at 42 °C. After hybridization, the membrane was washed with buffer 1 (2× SSC, 0.1% SDS) at room temperature for 15 minutes and then washed twice with buffer 2 (0.5× SSC, 0.1% SDS) at 55 °C for 15 minutes. The membranes were exposed overnight to phosphorimager plates, which were imaged on a Typhoon FLA 9500 biomolecular imager (GE Healthcare, Chicago, IL). Image quantification was performed using ImageQuant software to measure the intensity value of each telomere smear. The weighted centers of mass of density plot profiles were generated for each sample. Numeric values generated from the histograms were compared against values of a known molecular weight standard, to obtain the average telomere length.

### Telomere quantitative fluorescence in situ hybridization

HeLa and HCT116 cells were plated (200,000 cells/well in six-well plate) directly on coverslips. Cells were fixed with 4% paraformaldehyde, permeabilized with 0.5% Triton X-100, and dehydrated through graded alcohols prior to hybridization with a fluorescently labeled telomere leading strand PNA probe [5′-(CCCTAA)_3_−3′] (Cy5-TelC; PNA Bio, Newbury Park, CA) overnight at 25°C in a humidified chamber. Cells were washed twice with PNA wash buffer (70% formamide, 10 mM Tris-HCl pH 7.5, 1% BSA), counterstained with DAPI (1 μg/mL in PBS), and mounted onto glass slides with Fluoromount-G (Thermo Fisher Scientific, Waltham, MA). Images were captured using a Leica TCS SP8 STED confocal microscope (Light Microscopy Imaging Core, CWRU) and analyzed using the Leica Application Suite X (LAS X).

### Colony forming assay

Cells were treated with vehicle, AZT, or 5-MeCIdn for 3 days and then harvested. For each treatment group, 500 viable cells (HCT116, A549, U2OS, or WI-38) were seeded in three 35-mm plates and evenly dispersed. The cells were then grown in drug-free media for 11 days. The colonies were fixed (75% methanol and 25% acetic acid) for 15 minutes at room temperature and stained (0.05% crystal violet and 95.05% methanol) for 30 minutes at room temperature.

### Senescence-associated β-galactosidase staining

Cultured cells were plated into six-well dishes with a cover glass on it one week before staining. Cover glasses were collected and cells on it were fixed and stained as per the manufacturer's protocol (Senescence β-Galactosidase Staining Kit, Cell Signaling Technology no. 9860, Danvers, MA). Stained cells were imaged with a Leica SCN400 slide scanner. Ten random images were collected and cells counted with ImageJ 1.52e software. Staining was conducted 5 months after continual treatment with 100 μM 5-MeCIdR/DMSO or DMSO alone.

### Protein crystallization and data collection

The *T*. *castaneum* TERT protein was purified as described by Mitchel and colleagues [18]. The protein was dialyzed in 10 mM Tris-HCl, 100 mM KCl, 1 mM TCEP, pH 7.5 prior to crystallization trials. The ternary complex was prepared by adding to the 10 mg/mL dialyzed protein, 1.2 molar excess nucleic acid consisting of the putative RNA template and the complementary telomeric DNA (rCrUrGrArCrCrUrGrArCTTCGGTCAGGTCA—Integrated DNA Technologies), 1 mM 5-MeCITP, and 2.5 mM MgCl_2_. Crystals of the monoclinic space group P2_1_ were grown by the vapor diffusion sitting drop method. Drops were prepared by mixing one volume of the ternary complex with one volume of reservoir solution containing 0.1 M HEPES (pH 7.5), 8% PEG 8K, and 0.1 M KCl. The crystals were transferred into a cryosolution containing 0.1 M HEPES (pH 7.5), 8% PEG 8K, 30% PEG400, 0.1 M KCl, and 1 mM TCEP and flash frozen in liquid nitrogen. Data were collected from three crystals on a Rigaku MicroMax-007 HF rotating anode X-ray generator (wavelength 1.54178 Å) with VariMax optics and using a Saturn 944 HG CCD detector. The crystals were kept frozen with an Oxford Cryosystems Cobra system at 100 K during the data collection. The data were processed and scaled with XDS [54] (**S2 Table**). The coordinates and structure factors of the *T*. *castaneum* TERT-5-MeCITP complex have been deposited in the Protein Data Bank (PDB ID: 6E53).

### Structure determination and refinement

Phases were calculated by molecular replacement (MR) using PHASER [55] as implemented in PHENIX [56] using the *T*. *castaneum* TERT-hybrid structure (PDB ID: 3KYL) as a search model. The maps revealed clear *fo-fc* density for 5-MeCITP at 3.0 sigma contour level bound at the active site of the enzyme. Model building was carried out in COOT [57] and the model was refined using REFMAC5 [58] (**S2 Table**). The structure was refined to good stereochemistry with 92.1% and 7.9% of the residues in the “most favorable” and “additional allowed” of the Ramachandran plot, respectively.

### Molecular modeling and docking

*T*. *castaneum* TERT, HIV-1 RT, and Klenow fragment polymerase were loaded into Maestro (Schrodinger 2017–3 suite) using their PDB ID *(T*. *castaneum* TERT PDB ID: 3KYL; HIV-1 RT PDB ID: 3V4I; Klenow fragment polymerase PDB ID: 1KFD). Models were subjected to the Protein Preparation protocol with Workspace Structure Preprocessing, H-bond Assignment, and Restrained Minimization at default settings. Binding sites were defined by using the Receptor Grid Generation protocol, at default settings. dATP and other nucleoside analogues were modeled in with the software and docked at the respective binding sites using the Ligand Docking protocol, which allows for flexible docking using Extra Precision (XP) settings while also restricting docking to a reference position with tolerance of 0.10 Å. Reference position was designated as the location of ligand contained in their respective PDB files.

### Statistical analysis

All statistical analyses were carried out using Excel software. Unless otherwise noted, experiments were conducted in triplicate. Comparisons between groups were performed using two-tailed Student *t* test. Statistical significance was considered if *p*-values were <0.05. Individual *p*-values are indicated in figure legends. All results are expressed as means ± SD.

## Supporting information

S1 Table Physiochemical properties of indoly-2′-deoxynucleotide analogs used in this study. (DOCX)

S2 Table X-ray crystallography data collection and refinement statistics. (DOCX)

S1 Fig Artificial indolyl nucleotide analogs are not incorporated by telomerase into extended DNA products in vitro. The in vitro direct telomerase extension assay was performed to determine whether any of the nucleotide analogs could replace native dATP for telomerase-mediated extension of telomere DNA. Values on the left of the assay depict the number of nucleotides added by telomerase to a telomeric-mimic primer d(GGGTTA)_3_. The labels at the top define the nucleotide, either dATP or the artificial analog, that was used along with dTTP and dGTP for telomere extension in the reaction. dATP, deoxyadenosine triphosphate; dGTP, deoxyguanosine triphosphate; dTTP, deoxythymidine triphosphate; LC, loading control.
(TIF)

S2 Fig Determination of *K_1/2_* for dATP and dTTP in the telomerase extension assay. (**A**) Telomerase activity assay (top) in the presence of increasing concentrations of dATP (0, 0.1, 0.2, 0.3, 0.4, 0.5, 0.6, 0.7, 0.8, 0.9, 1, 5, and 10 μM) was fitted with a hyperbolic equation (bottom) to determine observed *K*_1/2_ value of dATP (*n* = 4). (**B**) Telomerase activity assay (top) in the presence of increasing concentrations of dTTP (0, 0.5, 1, 1.5, 2, 3, 3.5, 5, 10, and 100 μM) was fitted with a hyperbolic equation (bottom) to determine observed *K*_1/2_ value of dTTP (*n* = 4). (**C**) Telomerase activity assay reveals that increased extension times (0–120 mins) correlate with increased product. The red asterisk at 30 minutes indicates the pre-steady-state time point that was chosen for subsequent in vitro telomerase activity assays. dATP, deoxyadenosine triphosphate; dTTP, deoxythymidine triphosphate.
(TIF)

S3 Fig Inhibition of telomerase by AZT-TP. (**A**) An in vitro telomerase activity assay was performed with increasing concentrations of AZT-TP to calculate the *K’*_1/2_ under these experimental conditions and for comparison to that of 5-MeCITP. (**B**) AZT chemical structure and electron density of nucleobase surface potentials. For clarity, the triphosphate group was not included in the structure. AZT, azidothymidine; AZT-TP, AZT triphosphate; 5-MeCITP, 5-methylcarboxyl-indolyl-2′-deoxyriboside 5′-triphosphate.
(TIF)

S4 Fig Nucleotide 5-MeCITP competes with dATP in telomerase extension assays. (**A**) Direct telomerase extension assay with increasing concentrations of dATP (0, 0.2, 0.4, 0.8, 1, 4, 10, and 100 μM) in the absence (control, left side) and in the presence of 5-MeCITP (400 uM) (right side). (**B**) Quantification and hyperbolic fitting generated from the normalized G (dGTP) incorporation in the absence or presence of 5-MeCITP shown in panel A, with their respective *K*_1/2_ determined in the absence (black) or presence (green) of 5-MeCITP. Data associated with this figure can be found in the supplemental data file (S1 Data). dATP, deoxyadenosine triphosphate; dGTP, deoxyguanosine triphosphate; 5-MeCITP, 5-methylcarboxyl-indolyl-2′-deoxyriboside 5′-triphosphate.
(TIF)

S5 Fig The nucleobase of 5-MeCITP adopts a distinct binding mode compared with native dATP. (**A**) Overlay of 5-MeCITP bound (yellow) and dATP bound (blue) structures highlights differences in the binding pocket induced by the binding of 5-MeCITP (green) versus native dATP (blue). (**B**) Panel A rotated 90°. (**C**) View perpendicular to the nucleobase of superposed cognate dATP (blue) and 5-MeCITP (green). The superposition reveals that 5-MeCITP adopts an orientation in the TERT binding site, where the nucleobase is flipped 180° compared with that of dATP. dATP, deoxyadenosine triphosphate; TERT, telomerase reverse transcriptase; 5-MeCITP, 5-methylcarboxyl-indolyl-2′-deoxyriboside 5′-triphosphate.
(TIF)

S6 Fig Overlay of *Tribolium castaneum* TERT-RNA-DNA complex, *Homo sapiens* TERT, and *Tetrahymena thermophila* TERT shows structural conservation. (**A**) Overlay of *T*. *castaneum* TERT-RNA-DNA complex (green; PDB ID 3KYL), *H*. *sapiens* TERT (yellow; constructed by fitting of *T.t* TEN domain (PDB ID 2B2A) and *T.c* TERT, as independent domains, (PDB ID 3KYL) into the *H.s* telomerase cryo-EM map (EMD 7518)) and *T*. *thermophila* TERT (salmon; PDB ID 6D6V). The overlay indicates a conserved mechanism of RNA template and telomeric DNA binding. It is worth noting that in the *T*. *castaneum* TERT complex, several nucleotides at the 5′ end of the DNA and 3′ end of the RNA were introduced for crystallographic purposes and that portions of the nucleic acid do not make contacts with TERT. (**B**) Zoomed-in representation of panel A focused at the 3′ end of the telomeric DNA and where the 5-MeCITP binds. This view shows a high degree of conservation within this telomerase region across these three species. (**C**) Overlay of *T*. *castaneum* TERT-RNA-DNA complex (green) and *T*. *thermophila* TERT (salmon) active site. The main difference is that the loop K_481_HKEGS_486_ (*Tetrahymena* numbering; highlighted in red) in the RNA binding domain of the *T*. *thermophila* TERT structure includes more polar residues and is slightly displaced from the RNA template. EM, electron microscopy; TERT, telomerase reverse transcriptase; 5-MeCITP, 5-methylcarboxyl-indolyl-2′-deoxyriboside 5′-triphosphate.
(TIFF)

S7 Fig Nucleotide 5-MeCITP competes with native dNTPs to inhibit telomerase activity. Direct in vitro telomerase assay was performed with limiting concentrations of (**A**) dTTP, (**B**) dGTP, or (**C**) dATP and with increasing concentrations of 5-MeCITP. For each experiment, the identified native nucleotide was maintained at a limiting concentration near its calculated *K*_1/2_ value_._ Under each condition, telomerase activity was consistently inhibited with increasing 5-MeCITP concentrations. dATP, deoxyadenosine triphosphate; dGTP, deoxyguanosine triphosphate; dNTP, deoxynucleotide triphosphate; dTTP, deoxythymidine triphosphate; 5-MeCITP, 5-methylcarboxyl-indolyl-2′-deoxyriboside 5′-triphosphate.
(TIF)

S8 Fig Nucleotide 5-MeCIdR versus AZT treatment in telomerase-positive and -negative cell lines. Colony assay formation of (**A**) MIA-Pa-Ca-2 (telomerase positive), (**B**) HCT116 (telomerase positive), and (**C**) U2OS (telomerase negative) after 5 days of treatment reveals that AZT is associated with significantly reduced cell survival as compared with 5-MeCIdR. *N* = 3. Error bars indicate the standard deviation values of three replicates. Two-tailed Student *t* test, *p < 0.05, **p < 0.005. Data associated with this figure can be found in the supplemental data file (S1 Data). AZT, azidothymidine; 5-MeCIdR, cell-permeable nucleoside form of 5-MeCITP.
(TIF)

S9 Fig Nucleoside 5-MeCIdR leads to telomere shortening in telomerase-positive cells. Telomere length after 1 month’s treatment of 100 μM 5-MeCIdR in telomerase-positive HeLa cells (**A**) gets shorter with increasing PDs, while mock treated HeLa cells’ telomeres get longer over time. Numbers at the bottom of the Southern blots indicate the average telomere lengths. (**B**) Q-FISH shows higher fluorescent intensity for (**B**) HCT116 and (**C**) HeLa at Passage 1 (P1). Following treatment with 100 μM 5-MeCIdR (5-month HCT116, 1-month HeLa), telomere fluorescent intensity is considerably decreased when compared with mock induced conditions. Data associated with this figure can be found in the supplemental data file (S1 Data). PD, population doubling; Q-FISH, quantitative fluorescence in situ hybridization; 5-MeCIdR, cell-permeable form of 5-MeCITP.
(TIF)

S10 Fig Nucleoside 5-MeCIdR induces senescence in telomerase-positive cells A549, MIA-Pa-Ca-2, and HCT116. SA-β-gal, a senescence marker, was used for staining of telomerase-positive cells A549 MIA-Pa-Ca-2 and HCT116 after treatment with 100 μM 5-MeCIdR or DMSO (control) for 5 months. (**A**) Photographic images (magnification 20×) and (**B**) quantitation of the data. **p < 0.005, ***p < 0.0001. Data associated with this figure can be found in the supplemental data file (S1 Data). 5-MeCIdR, cell-permeable nucleoside form of 5-MeCITP.
(TIFF)

S11 Fig Predictive models of interactions of the non-native nucleotide analogs docked into the *Tribolium castaneum* TERT structure. In silico models of dATP and nucleoside analogs (**A**. dATP, **B**. 6-NITP, **C**. 5-MeITP, **D**. 5-MeCITP, **E**. 5-FITP, **F**. 5-EyITP, **G**. 5-CITP, **H**. 5-AITP, **I**. 4-NITP) docked into the binding site of the *T*. *castaneum* TERT structure. Models were generated with Maestro (Schrodinger 2017–3) using Ligand Docking in the *T*. *castaneum* TERT structure; PDB ID: 3KYL. Generated models predict that the side chain of 5-MeCITP exclusively forms favorable interactions with a hydrophobic pocket of TERT that is formed by the unique motifs 1 and 2 and motif T of TERT proteins. dATP, deoxyadenosine triphosphate; TERT, telomerase reverse transcriptase; 4-NITP, 4-nitroindolyl-2′-deoxynucleoside 5′-triphosphate; 6-NITP, 6-nitroindolyl-2′-deoxynucleoside 5′-triphosphate; 5-AITP, 5-aminoindolyl-2′-deoxyriboside 5′-triphosphate; 5-CITP, 5-carboxylindolyl-2′-deoxyriboside 5′-triphosphate; 5-EyITP, 5-ethyleneindolyl-2′-deoxyriboside 5′-triphosphate; 5-FITP, 5-fluoroindolyl-2′-deoxyriboside 5′-triphosphate; 5-MeCITP, 5-methylcarboxyl-indolyl-2′-deoxyriboside 5′-triphosphate; 5-MeITP, 5-methylindolyl-2′-deoxyriboside 5′-triphosphate.
(TIFF)

S12 Fig Interactions of 5-MeCITP with *Tc*TERT compared with predicted interactions of 5-MeCITP with HIV-RT and *Escherichia coli* Klenow fragment DNA polymerase I. Comparison of 5-MeCITP in the binding site of *T*. *castaneum* TERT (**A**), HIV-RT (**B**), and Klenow fragment DNA polymerase I (**C**). The overall conserved domains of the polymerases’ structures are depicted, with the finger domain shown in orange, the palm domain colored yellow, and the thumb domain in red. The TERT-specific TRBD is covered blue in panel A. The RT/TERT-specific motif 1 and motif 2 are identified at the bottom as 1 and 2 in orange, respectively, in panels A and B. The TERT-specific motif T is identified as T with blue borders in panel A. At the top of each panel, the domain organization of *T*. *castaneum* TERT, HIV-RT, and Klenow fragment DNA polymerase I are shown color coded as previously stated. In the middle row, the ribbon representation of the three-dimensional structures of each polymerase with 5-MeCITP modeled into the active site is shown. In the bottom row, a close-up view of the modeled 5-MeCITP is shown along with the surface area of the active site for each polymerase. Docking of 5-MeCITP, and binding site surface, were created in Maestro (Schrodinger 2017–3). HIV-RT, human immunodeficiency virus reverse transcriptase; *Tc*TERT, *T*. *castaneum* telomerase reverse transcriptase; TERT, telomerase reverse transcriptase; TRBD, telomerase RNA-binding domain; 5-MeCITP, 5-methylcarboxyl-indolyl-2′-deoxyriboside 5′-triphosphate.
(TIF)

S1 Data Data. (XLSX)

## Abbreviations

ALT: alternative lengthening of telomeres
AZT: azidothymidine
AZT-TP: AZT triphosphate
dATP: deoxyadenosine triphosphate
dG: deoxyguanosine
dGTP: deoxyguanosine triphosphate
dNTP: deoxynucleoside triphosphate
DMEM: Dulbecco’s Modified Eagle Medium
dTTP: deoxythymidine triphosphate
FBS: fetal bovine serum
*K’*_1/2_: apparent rate constant
*K*_1/2_: relative rate constant
MR: molecular replacement
NRTI: nucleoside reverse transcriptase inhibitor
RT: reverse transcriptase
ssDNA: single stranded DNA
TBE: template boundary element
TERT: telomerase reverse transcriptase
TR: telomerase RNA template
TRBD: telomerase RNA-binding domain
TRF: telomere restriction fragment
4-NITP: 4-nitroindolyl-2′-deoxyriboside 5′-triphosphate
5-CITP: 5-carboxylindolyl-2′-deoxyriboside 5′-triphosphate
5-EyITP: 5-ethyleneindolyl-2′-deoxyriboside 5′-triphosphate
5-FITP: 5-fluoroindolyl-2′-deoxyriboside 5′-triphosphate
5-MeCIdR: cell-permeable nucleoside form of 5-MeCITP
5-MeCITP: 5-methylcarboxyl-indolyl-2′-deoxyriboside 5′-triphosphate
5-MeITP: 5-methylindolyl-2′-deoxyriboside 5′-triphosphate
6-NITP: 6-nitroindolyl-2′-deoxyriboside 5′-triphosphate
