# Supplementary material for: A non-natural nucleotide uses a specific pocket to selectively inhibit telomerase activity
Source: PLoS Biol. 2019 Apr 5;17(4):e3000204. doi: 10.1371/journal.pbio.3000204 (PMC6469803; doi:10.1371/journal.pbio.3000204)
Supplement: S1 Table — (DOCX) [file pbio.3000204.s001.docx]

**S1** **Table.** Physiochemical properties of indoly-2’-deoxynucleotide analogs used in this study.

| **Nucleotide** | **Full name** | **Surface Area (Å^2^)** | **cLog*P*** |
| --- | --- | --- | --- |
| dATP | 2′-deoxyadenosine 5′-triphosphate | 144 | -1.45 |
| 4-NITP | 4-nitroindolyl-2'-deoxynucleoside 5'-triphosphate | 172 | -0.07 |
| 6-NITP | 6-nitroindolyl-2'-deoxynucleoside 5'-triphosphate | 175 | -0.07 |
| 5-FITP | 5-fluoroindolyl-2'-deoxyriboside 5'-triphosphate | 153 | 1.80 |
| 5-MeITP | 5-methylindolyl-2'-deoxyriboside 5'-triphosphate | 167 | 2.12 |
| 5-MeCITP | 5-methylcarboxyl-indolyl-2'-deoxyriboside 5'-triphosphate | 200 | 1.46 |
| 5-CITP | 5-carboxylindolyl-2'-deoxyriboside 5'-triphosphate | 178 | 1.19 |
| 5-EyITP | 5-ethyleneindolyl-2'-deoxyriboside 5'-triphosphate | 181 | 2.27 |
| 5-AITP | 5-aminoindolyl-2'-deoxyriboside 5'-triphosphate | 161 | 0.83 |

Surface area (relative size of the nucleobase) and cLog*P* (hydrophobicity) of each compound were calculated using Spartan 08 software.
