# Supplementary material for: A non-natural nucleotide uses a specific pocket to selectively inhibit telomerase activity
Source: PLoS Biol. 2019 Apr 5;17(4):e3000204. doi: 10.1371/journal.pbio.3000204 (PMC6469803; doi:10.1371/journal.pbio.3000204)
Supplement: S2 Table — (DOCX) [file pbio.3000204.s002.docx]

**S2 Table.** X-ray crystallography data collection and refinement statistics

|  | **TERT complex** |
| --- | --- |
| **Data collection** |  |
| Space group | P2_1_ |
| Cell dimensions |  |
| *a*, *b*, *c* (Å) | 80.0 52.1 100.5 |
| *α, β, γ* (°) | 90 98.5 90 |
| Resolution (Å) | 20-2.8 (2.95-2.80)* |
| CC(1/2) | 99.9 (55.5) |
| *I* / σ*I* | 12.1 (1.1) |
| Completeness (%) | 98.8 (99.3) |
| Redundancy | 20 (18) |
|  |  |
| **Refinement** |  |
| Resolution (Å) | 20-2.8 |
| No. reflections | 19145 |
| *R*_work_ / *R*_free_ | 24.2/29.3 |
| No. atoms | 5495 |
| Protein | 4987 |
| Ligand/ion | 509/2 |
| *B*-factors |  |
| Protein | 59 |
| Ligand/ion | 77 |
| R.m.s. deviations |  |
| Bond lengths (Å) | 0.004 |
| Bond angles (°) | 0.840 |

* Number of crystals used - three. *Values in parentheses are for highest-resolution shell.
