# Supplementary figures and images for: A non-natural nucleotide uses a specific pocket to selectively inhibit telomerase activity
Source: PLoS Biol. 2019 Apr 5;17(4):e3000204. doi: 10.1371/journal.pbio.3000204 (PMC6469803; doi:10.1371/journal.pbio.3000204)

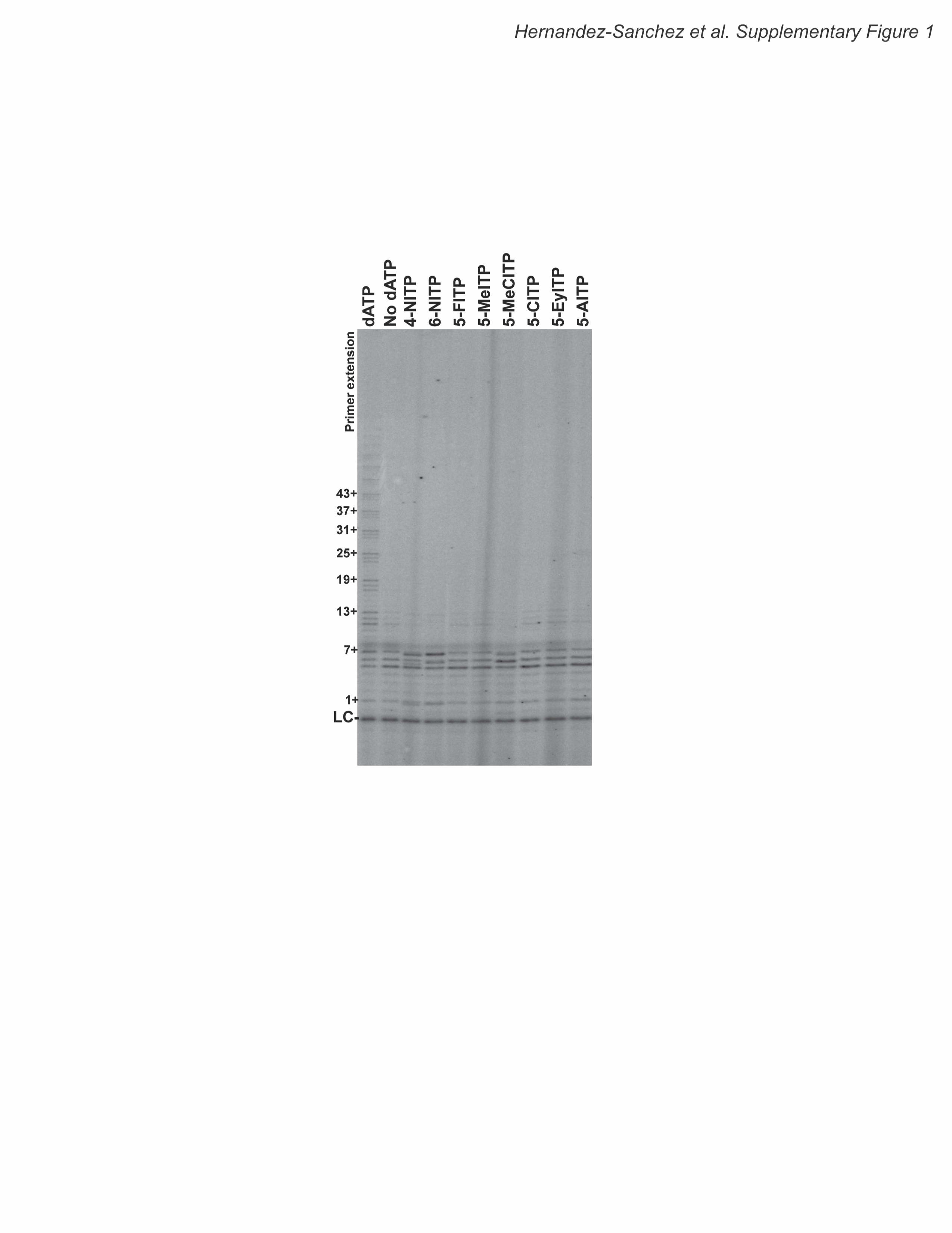

Supplement: S1 Fig — The in vitro direct telomerase extension assay was performed to determine whether any of the nucleotide analogs could replace native dATP for telomerase-mediated extension of telomere DNA. Values on the left of the assay depict the number of nucleotides added by telomerase to a telomeric-mimic primer d(GGGTTA)3. The labels at the top define the nucleotide, either dATP or the artificial analog, that was used along with dTTP and dGTP for telomere extension in the reaction. dATP, deoxyadenosine triphosphate; dGTP, deoxyguanosine triphosphate; dTTP, deoxythymidine triphosphate; LC, loading control. (TIF) [file pbio.3000204.s003.tif]

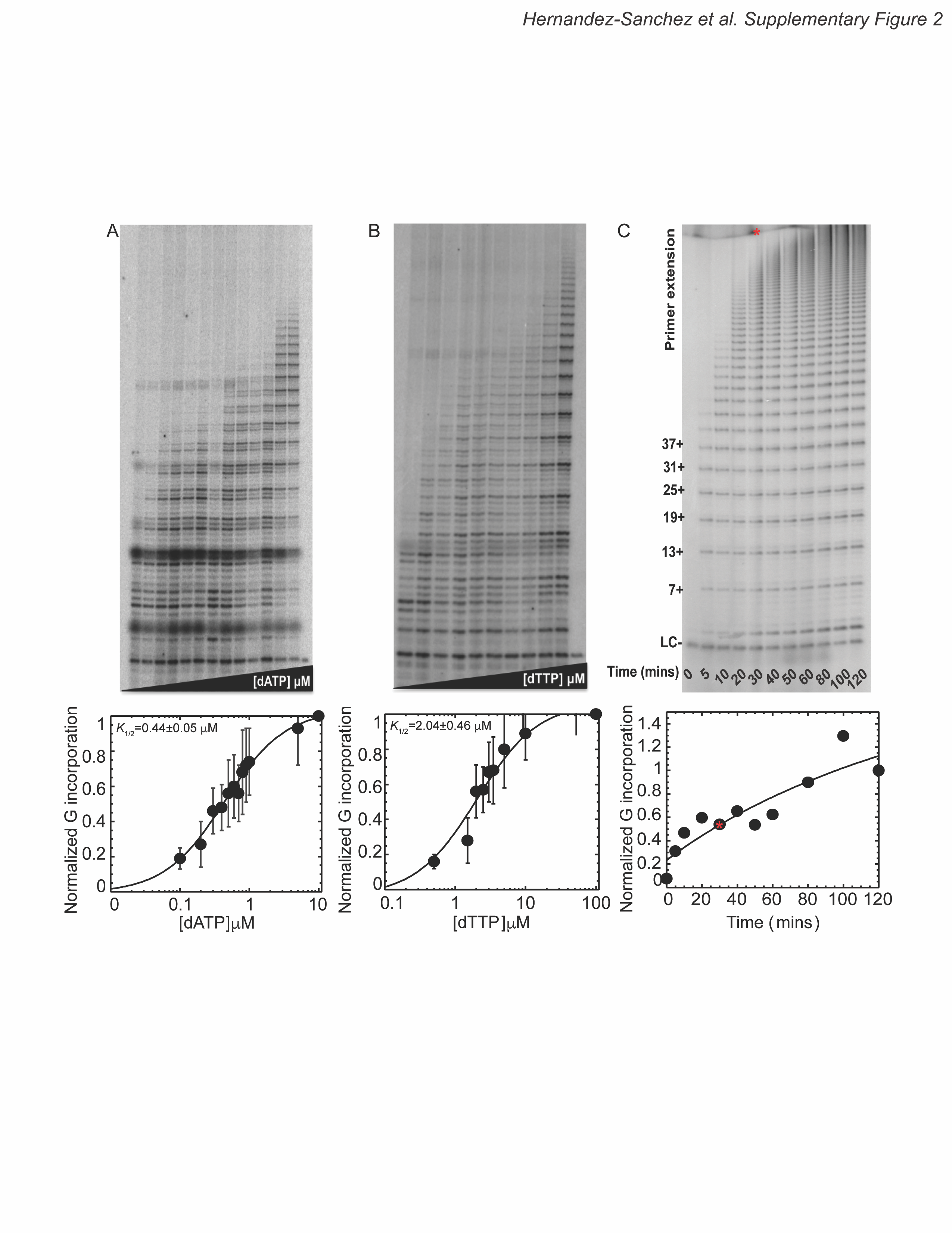

Supplement: S2 Fig — (A) Telomerase activity assay (top) in the presence of increasing concentrations of dATP (0, 0.1, 0.2, 0.3, 0.4, 0.5, 0.6, 0.7, 0.8, 0.9, 1, 5, and 10 μM) was fitted with a hyperbolic equation (bottom) to determine observed K1/2 value of dATP (n = 4). (B) Telomerase activity assay (top) in the presence of increasing concentrations of dTTP (0, 0.5, 1, 1.5, 2, 3, 3.5, 5, 10, and 100 μM) was fitted with a hyperbolic equation (bottom) to determine observed K1/2 value of dTTP (n = 4). (C) Telomerase activity assay reveals that increased extension times (0–120 mins) correlate with increased product. The red asterisk at 30 minutes indicates the pre-steady-state time point that was chosen for subsequent in vitro telomerase activity assays. dATP, deoxyadenosine triphosphate; dTTP, deoxythymidine triphosphate. (TIF) [file pbio.3000204.s004.tif]

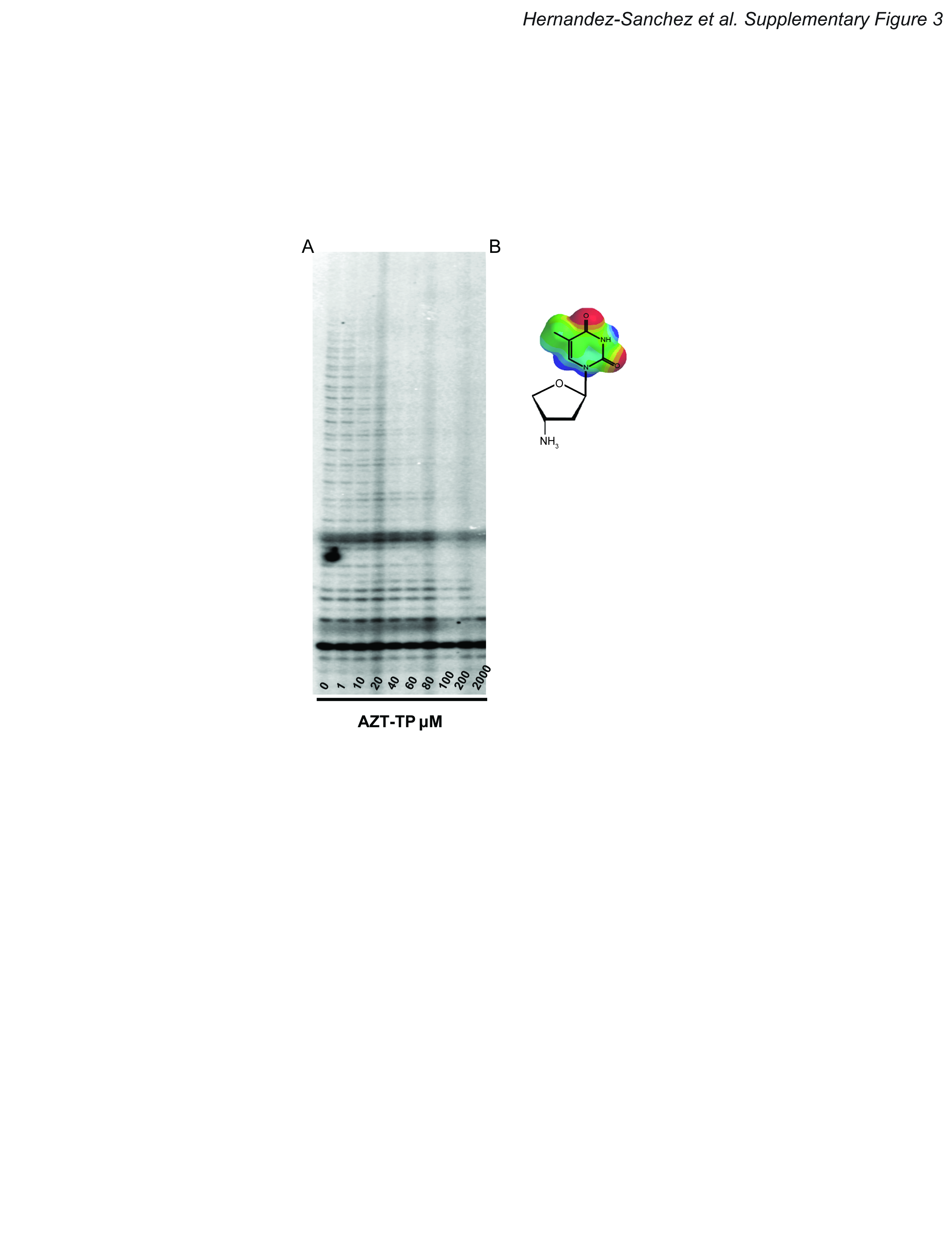

Supplement: S3 Fig — (A) An in vitro telomerase activity assay was performed with increasing concentrations of AZT-TP to calculate the K’1/2 under these experimental conditions and for comparison to that of 5-MeCITP. (B) AZT chemical structure and electron density of nucleobase surface potentials. For clarity, the triphosphate group was not included in the structure. AZT, azidothymidine; AZT-TP, AZT triphosphate; 5-MeCITP, 5-methylcarboxyl-indolyl-2′-deoxyriboside 5′-triphosphate. (TIF) [file pbio.3000204.s005.tif]

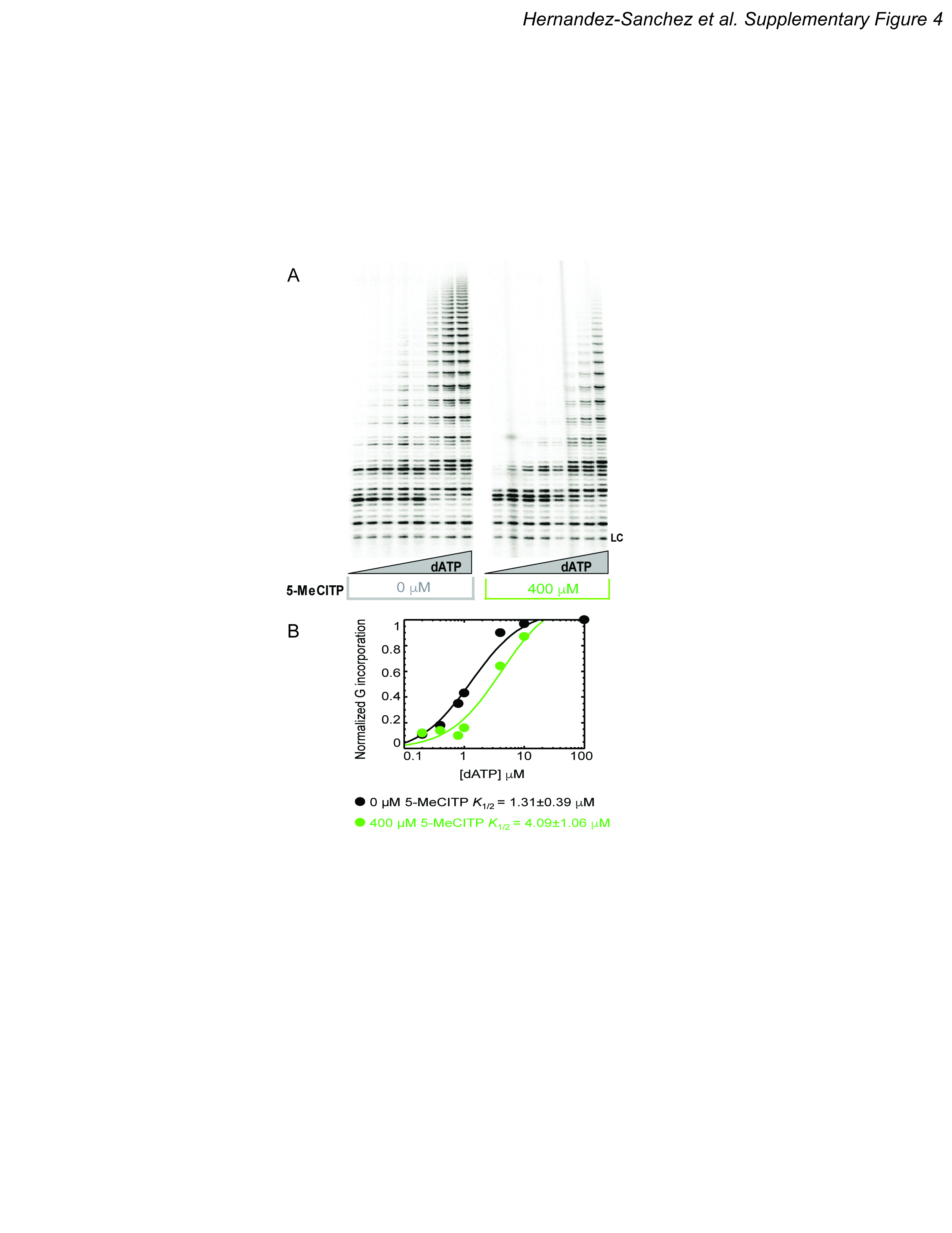

Supplement: S4 Fig — (A) Direct telomerase extension assay with increasing concentrations of dATP (0, 0.2, 0.4, 0.8, 1, 4, 10, and 100 μM) in the absence (control, left side) and in the presence of 5-MeCITP (400 uM) (right side). (B) Quantification and hyperbolic fitting generated from the normalized G (dGTP) incorporation in the absence or presence of 5-MeCITP shown in panel A, with their respective K1/2 determined in the absence (black) or presence (green) of 5-MeCITP. Data associated with this figure can be found in the supplemental data file (S1 Data). dATP, deoxyadenosine triphosphate; dGTP, deoxyguanosine triphosphate; 5-MeCITP, 5-methylcarboxyl-indolyl-2′-deoxyriboside 5′-triphosphate. (TIF) [file pbio.3000204.s006.tif]

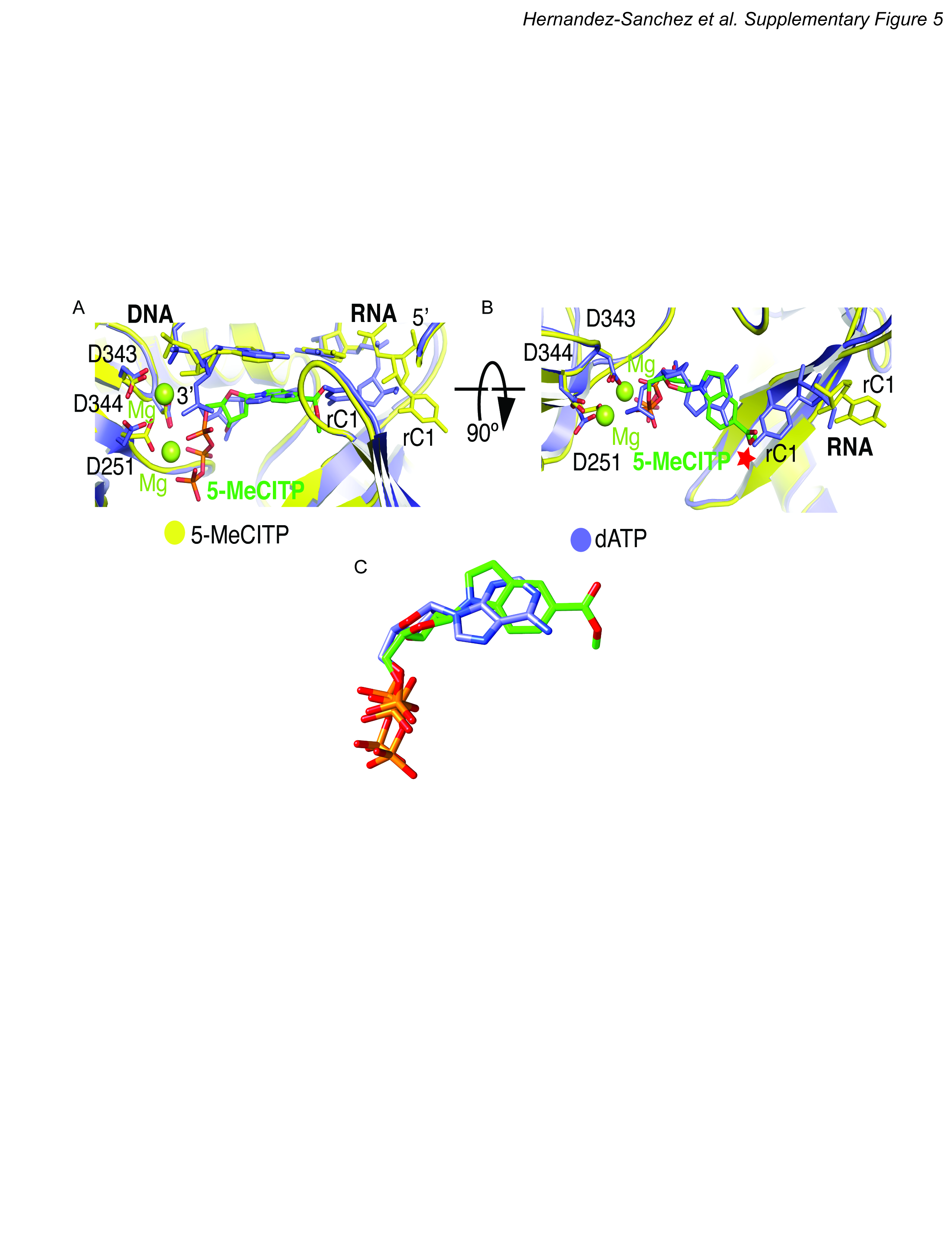

Supplement: S5 Fig — (A) Overlay of 5-MeCITP bound (yellow) and dATP bound (blue) structures highlights differences in the binding pocket induced by the binding of 5-MeCITP (green) versus native dATP (blue). (B) Panel A rotated 90°. (C) View perpendicular to the nucleobase of superposed cognate dATP (blue) and 5-MeCITP (green). The superposition reveals that 5-MeCITP adopts an orientation in the TERT binding site, where the nucleobase is flipped 180° compared with that of dATP. dATP, deoxyadenosine triphosphate; TERT, telomerase reverse transcriptase; 5-MeCITP, 5-methylcarboxyl-indolyl-2′-deoxyriboside 5′-triphosphate. (TIF) [file pbio.3000204.s007.tif]

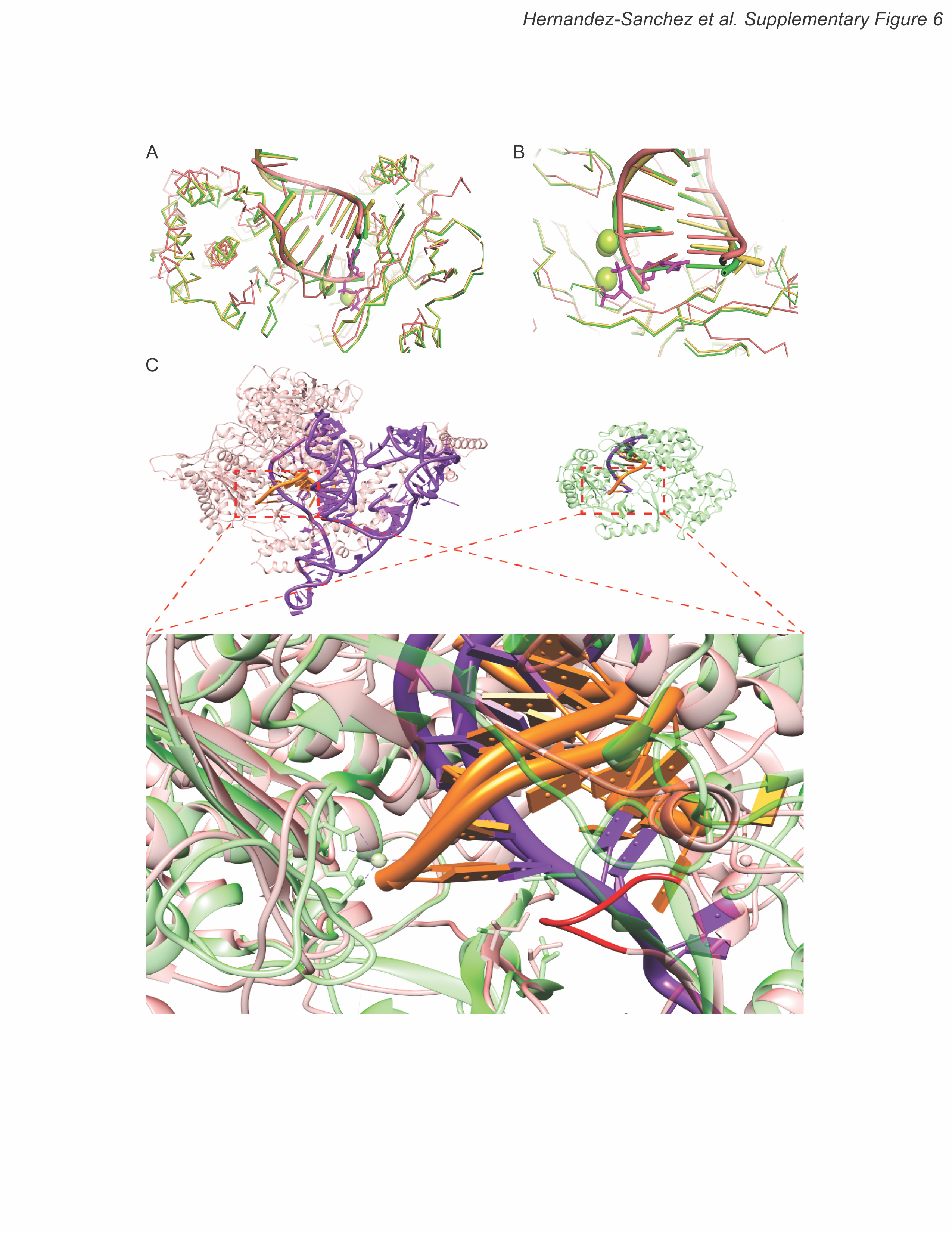

Supplement: S6 Fig — (A) Overlay of T. castaneum TERT-RNA-DNA complex (green; PDB ID 3KYL), H. sapiens TERT (yellow; constructed by fitting of T.t TEN domain (PDB ID 2B2A) and T.c TERT, as independent domains, (PDB ID 3KYL) into the H.s telomerase cryo-EM map (EMD 7518)) and T. thermophila TERT (salmon; PDB ID 6D6V). The overlay indicates a conserved mechanism of RNA template and telomeric DNA binding. It is worth noting that in the T. castaneum TERT complex, several nucleotides at the 5′ end of the DNA and 3′ end of the RNA were introduced for crystallographic purposes and that portions of the nucleic acid do not make contacts with TERT. (B) Zoomed-in representation of panel A focused at the 3′ end of the telomeric DNA and where the 5-MeCITP binds. This view shows a high degree of conservation within this telomerase region across these three species. (C) Overlay of T. castaneum TERT-RNA-DNA complex (green) and T. thermophila TERT (salmon) active site. The main difference is that the loop K481HKEGS486 (Tetrahymena numbering; highlighted in red) in the RNA binding domain of the T. thermophila TERT structure includes more polar residues and is slightly displaced from the RNA template. EM, electron microscopy; TERT, telomerase reverse transcriptase; 5-MeCITP, 5-methylcarboxyl-indolyl-2′-deoxyriboside 5′-triphosphate. (TIFF) [file pbio.3000204.s008.tiff]

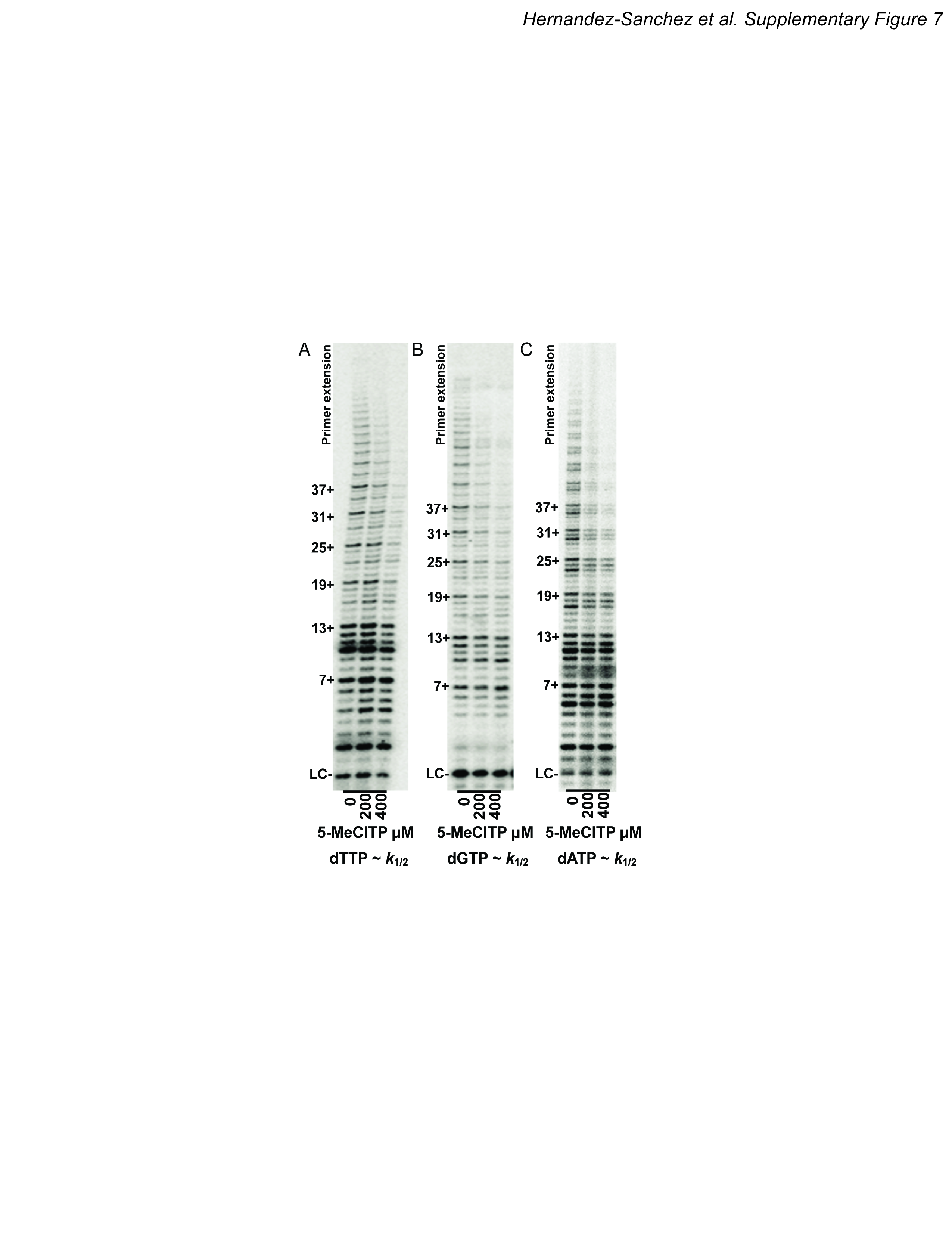

Supplement: S7 Fig — Direct in vitro telomerase assay was performed with limiting concentrations of (A) dTTP, (B) dGTP, or (C) dATP and with increasing concentrations of 5-MeCITP. For each experiment, the identified native nucleotide was maintained at a limiting concentration near its calculated K1/2 value. Under each condition, telomerase activity was consistently inhibited with increasing 5-MeCITP concentrations. dATP, deoxyadenosine triphosphate; dGTP, deoxyguanosine triphosphate; dNTP, deoxynucleotide triphosphate; dTTP, deoxythymidine triphosphate; 5-MeCITP, 5-methylcarboxyl-indolyl-2′-deoxyriboside 5′-triphosphate. (TIF) [file pbio.3000204.s009.tif]

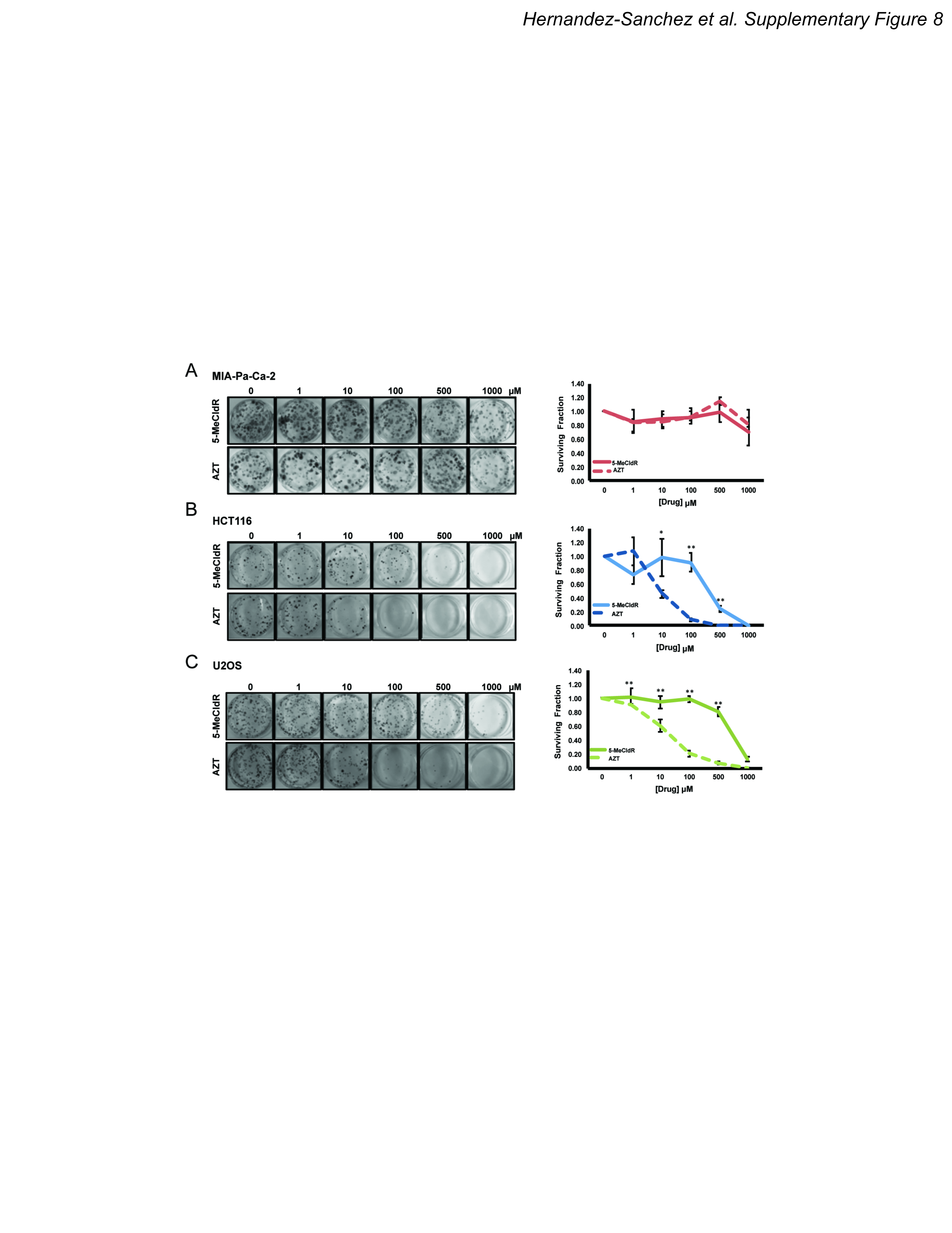

Supplement: S8 Fig — Colony assay formation of (A) MIA-Pa-Ca-2 (telomerase positive), (B) HCT116 (telomerase positive), and (C) U2OS (telomerase negative) after 5 days of treatment reveals that AZT is associated with significantly reduced cell survival as compared with 5-MeCIdR. N = 3. Error bars indicate the standard deviation values of three replicates. Two-tailed Student t test, *p < 0.05, **p < 0.005. Data associated with this figure can be found in the supplemental data file (S1 Data). AZT, azidothymidine; 5-MeCIdR, cell-permeable nucleoside form of 5-MeCITP. (TIF) [file pbio.3000204.s010.tif]

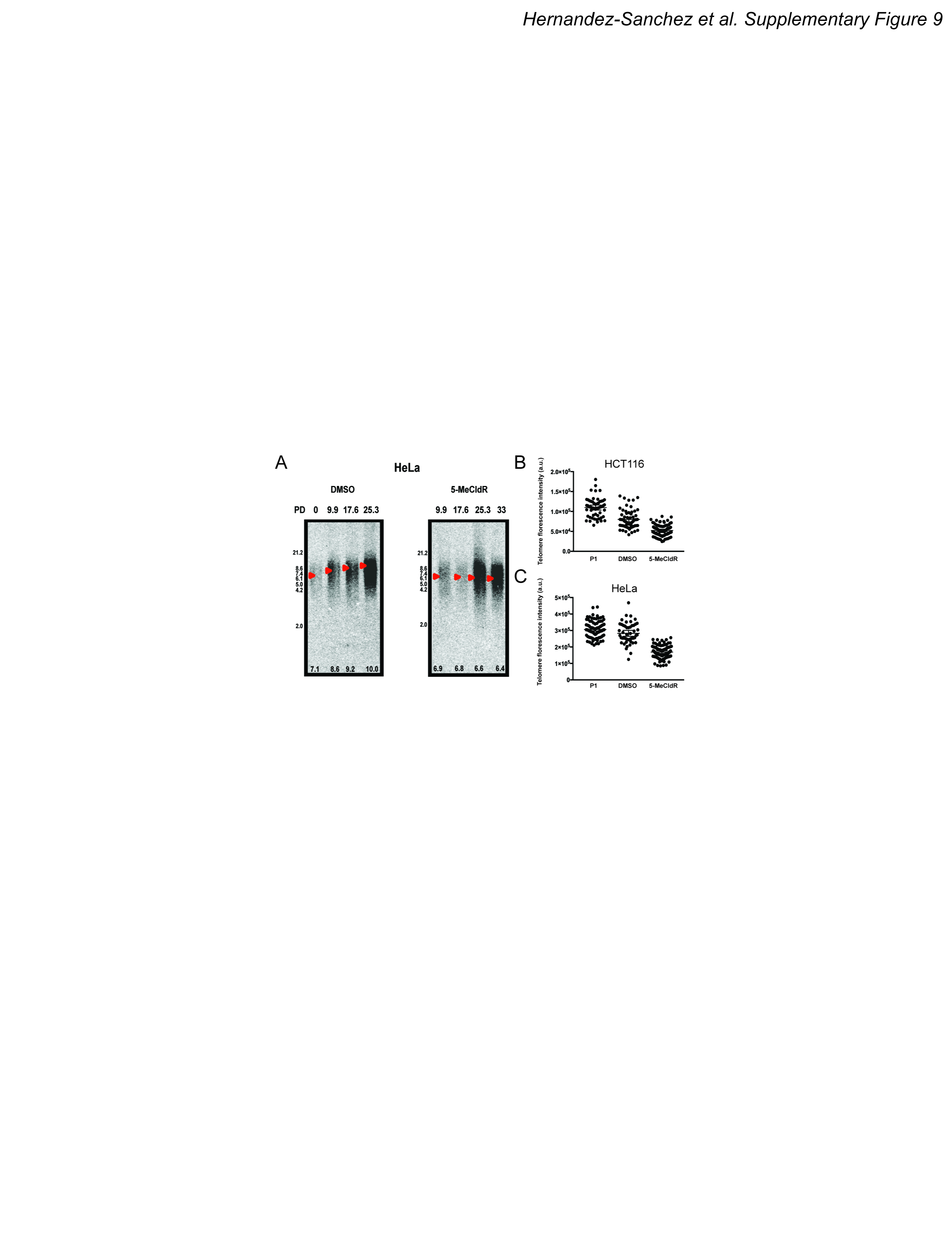

Supplement: S9 Fig — Telomere length after 1 month’s treatment of 100 μM 5-MeCIdR in telomerase-positive HeLa cells (A) gets shorter with increasing PDs, while mock treated HeLa cells’ telomeres get longer over time. Numbers at the bottom of the Southern blots indicate the average telomere lengths. (B) Q-FISH shows higher fluorescent intensity for (B) HCT116 and (C) HeLa at Passage 1 (P1). Following treatment with 100 μM 5-MeCIdR (5-month HCT116, 1-month HeLa), telomere fluorescent intensity is considerably decreased when compared with mock induced conditions. Data associated with this figure can be found in the supplemental data file (S1 Data). PD, population doubling; Q-FISH, quantitative fluorescence in situ hybridization; 5-MeCIdR, cell-permeable form of 5-MeCITP. (TIF) [file pbio.3000204.s011.tif]

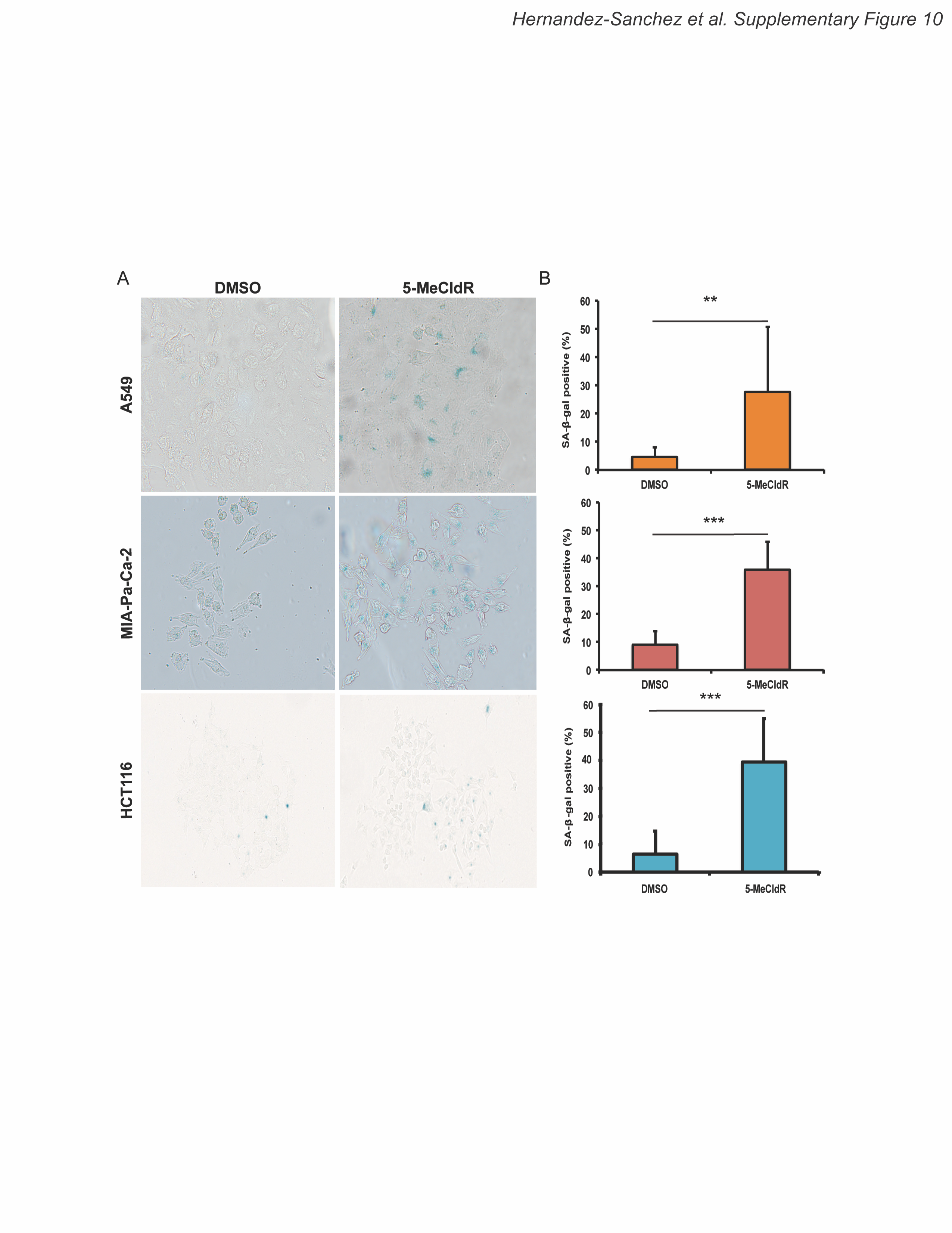

Supplement: S10 Fig — SA-β-gal, a senescence marker, was used for staining of telomerase-positive cells A549 MIA-Pa-Ca-2 and HCT116 after treatment with 100 μM 5-MeCIdR or DMSO (control) for 5 months. (A) Photographic images (magnification 20×) and (B) quantitation of the data. **p < 0.005, ***p < 0.0001. Data associated with this figure can be found in the supplemental data file (S1 Data). 5-MeCIdR, cell-permeable nucleoside form of 5-MeCITP. (TIFF) [file pbio.3000204.s012.tiff]

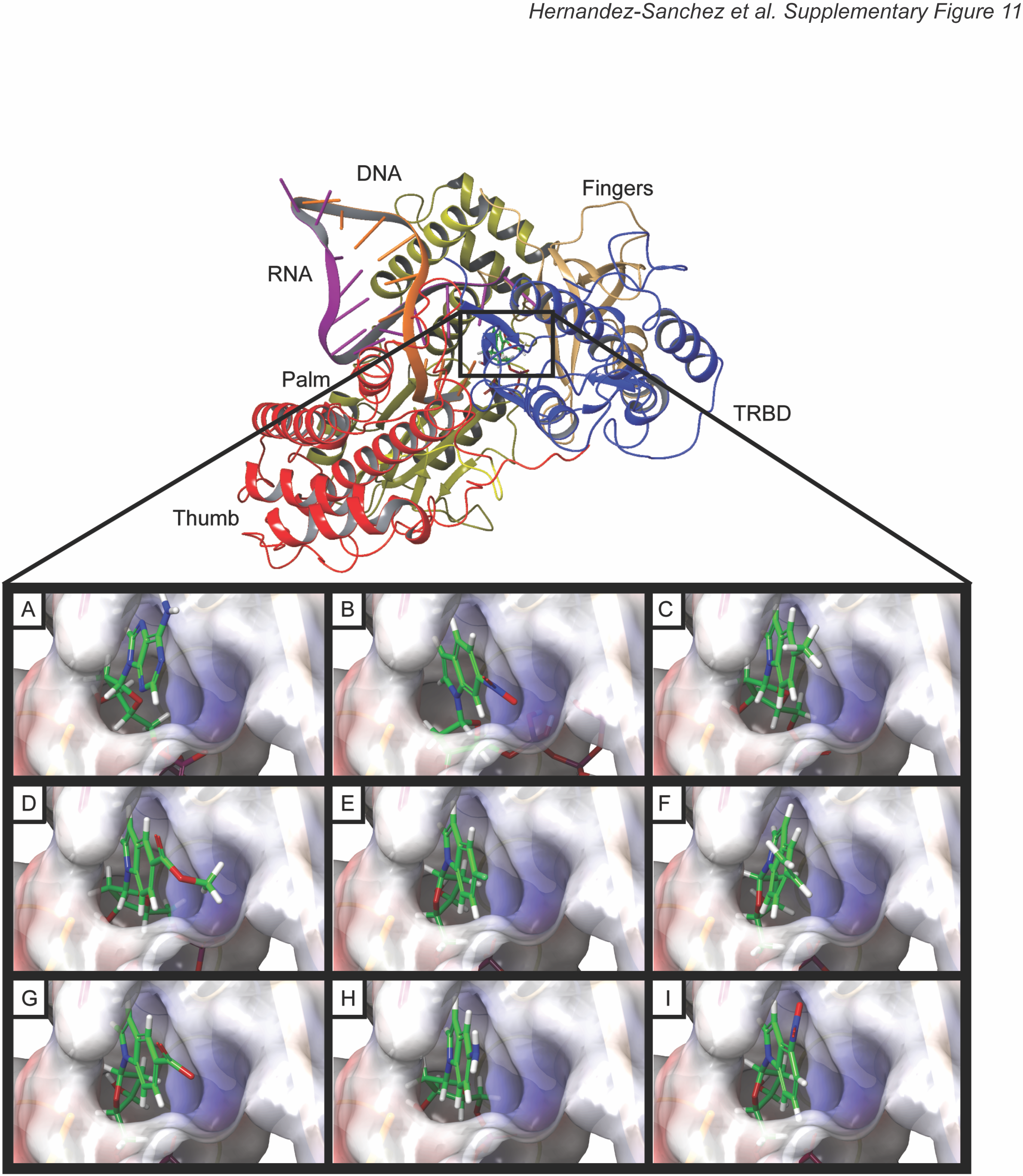

Supplement: S11 Fig — In silico models of dATP and nucleoside analogs (A. dATP, B. 6-NITP, C. 5-MeITP, D. 5-MeCITP, E. 5-FITP, F. 5-EyITP, G. 5-CITP, H. 5-AITP, I. 4-NITP) docked into the binding site of the T. castaneum TERT structure. Models were generated with Maestro (Schrodinger 2017–3) using Ligand Docking in the T. castaneum TERT structure; PDB ID: 3KYL. Generated models predict that the side chain of 5-MeCITP exclusively forms favorable interactions with a hydrophobic pocket of TERT that is formed by the unique motifs 1 and 2 and motif T of TERT proteins. dATP, deoxyadenosine triphosphate; TERT, telomerase reverse transcriptase; 4-NITP, 4-nitroindolyl-2′-deoxynucleoside 5′-triphosphate; 6-NITP, 6-nitroindolyl-2′-deoxynucleoside 5′-triphosphate; 5-AITP, 5-aminoindolyl-2′-deoxyriboside 5′-triphosphate; 5-CITP, 5-carboxylindolyl-2′-deoxyriboside 5′-triphosphate; 5-EyITP, 5-ethyleneindolyl-2′-deoxyriboside 5′-triphosphate; 5-FITP, 5-fluoroindolyl-2′-deoxyriboside 5′-triphosphate; 5-MeCITP, 5-methylcarboxyl-indolyl-2′-deoxyriboside 5′-triphosphate; 5-MeITP, 5-methylindolyl-2′-deoxyriboside 5′-triphosphate. (TIFF) [file pbio.3000204.s013.tiff]

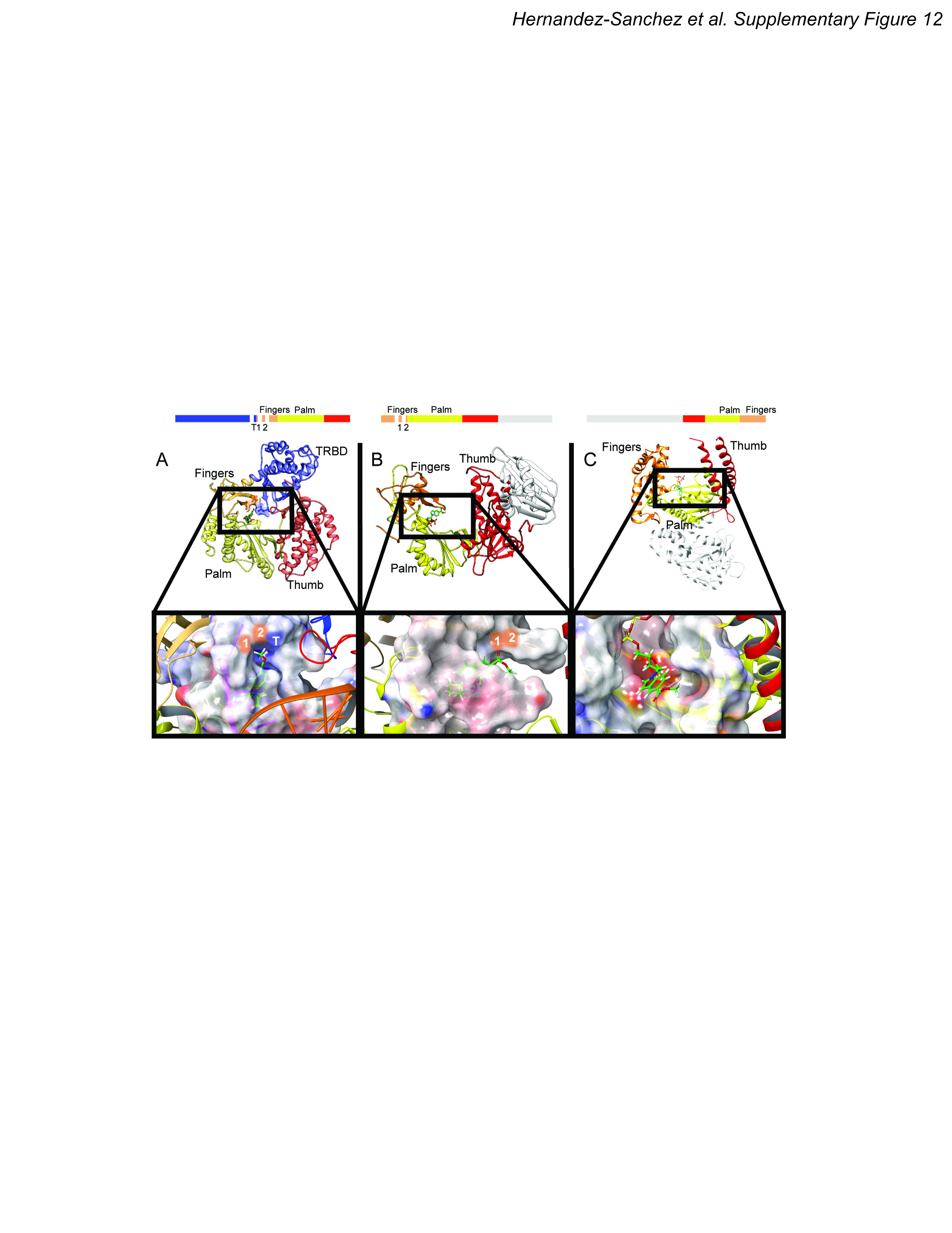

Supplement: S12 Fig — Comparison of 5-MeCITP in the binding site of T. castaneum TERT (A), HIV-RT (B), and Klenow fragment DNA polymerase I (C). The overall conserved domains of the polymerases’ structures are depicted, with the finger domain shown in orange, the palm domain colored yellow, and the thumb domain in red. The TERT-specific TRBD is covered blue in panel A. The RT/TERT-specific motif 1 and motif 2 are identified at the bottom as 1 and 2 in orange, respectively, in panels A and B. The TERT-specific motif T is identified as T with blue borders in panel A. At the top of each panel, the domain organization of T. castaneum TERT, HIV-RT, and Klenow fragment DNA polymerase I are shown color coded as previously stated. In the middle row, the ribbon representation of the three-dimensional structures of each polymerase with 5-MeCITP modeled into the active site is shown. In the bottom row, a close-up view of the modeled 5-MeCITP is shown along with the surface area of the active site for each polymerase. Docking of 5-MeCITP, and binding site surface, were created in Maestro (Schrodinger 2017–3). HIV-RT, human immunodeficiency virus reverse transcriptase; TcTERT, T. castaneum telomerase reverse transcriptase; TERT, telomerase reverse transcriptase; TRBD, telomerase RNA-binding domain; 5-MeCITP, 5-methylcarboxyl-indolyl-2′-deoxyriboside 5′-triphosphate. (TIF) [file pbio.3000204.s014.tif]
